# Energetics of Excitatory and Inhibitory Neurotransmission in Aluminum Chloride Model of Alzheimer’s Disease: Reversal of Behavioral and Metabolic Deficits by Rasa Sindoor

**DOI:** 10.3389/fnmol.2017.00323

**Published:** 2017-10-17

**Authors:** Kamal Saba, Niharika Rajnala, Pandichelvam Veeraiah, Vivek Tiwari, Rohit K. Rana, Subhash C. Lakhotia, Anant B. Patel

**Affiliations:** ^1^NMR Microimaging and Spectroscopy, CSIR-Centre for Cellular and Molecular Biology, Habsiguda, India; ^2^CSIR-Indian Institute of Chemical Technology, Tarnaka, India; ^3^Cytogenetics Laboratory, Department of Zoology, Banaras Hindu University, Varanasi, India

**Keywords:** glutamate, GABA, neurodegeneration, neurotransmitter cycle, neuron–glia trafficking, nuclear magnetic resonance spectroscopy

## Abstract

Alzheimer’s disease (AD) is an age-related neurodegenerative disorder, characterized by progressive loss of cognitive functions and memory. Excessive intake of aluminum chloride in drinking water is associated with amyloid plaques and neurofibrillary tangles in the brain, which are the hallmark of AD. We have evaluated brain energy metabolism in aluminum chloride (AlCl_3_) mouse model of AD. In addition, effectiveness of Rasa Sindoor (RS), a formulation used in Indian Ayurvedic medicine, for alleviation of symptoms of AD was evaluated. Mice were administered AlCl_3_ (40 mg/kg) intraperitoneally once a day for 60 days. The memory of mice was measured using Morris Water Maze test. The ^13^C labeling of brain amino acids was measured *ex vivo* in tissue extracts using ^1^H-[^13^C]-NMR spectroscopy with timed infusion of [1,6-^13^C_2_]glucose. The ^13^C turnover of brain amino acids was analyzed using a three-compartment metabolic model to derive the neurotransmitter cycling and TCA cycle rates associated with glutamatergic and GABAergic pathways. Exposure of AlCl_3_ led to reduction in memory of mice. The glutamatergic and GABAergic neurotransmitter cycling and glucose oxidation were found to be reduced in the cerebral cortex, hippocampus, and striatum following chronic AlCl_3_ treatment. The perturbation in metabolic rates was highest in the cerebral cortex. However, reduction in metabolic fluxes was higher in hippocampus and striatum following one month post AlCl_3_ treatment. Most interestingly, oral administration of RS (2 g/kg) restored memory as well as the energetics of neurotransmission in mice exposed to AlCl_3_. These data suggest therapeutic potential of RS to manage cognitive functions and memory in preclinical AD.

## Introduction

Alzheimer’s disease (AD) is the most common neurodegenerative disorder, accounting for majority of dementia worldwide (Blennow et al., 2006). It is characterized by progressive loss in memory and cognitive functions. The hallmark of disease is the presence of senile plaques and neurofibrillary tangles in the brain (Whitehouse et al., 1982). The neurodegeneration in AD starts much before the appearance of the first clinical symptoms. The early clinical phase, the amnestic mild cognitive impairment has neuropathological features intermediate between those of normal aging and AD, in which Tau deposits are abundant in the entorhinal cortex and hippocampus. Spreading of high amyloid load and Tau pathology to the entire cortical regions marks the later stages of AD. Deficits in numerous neurotransmitters increase with progression of disease (Selkoe, 1998; Hardy and Selkoe, 2002). Pathogenesis of AD is complex and remains elusive. Mutations in different genes like amyloid β-protein precursor (AβPP), presenilin-1 (PS1), presenilin-2 (PS2), and Tau have been identified as genetic determinants for AD (Selkoe, 2001). This led to generation of different transgenic models to understand mechanism and pathology of AD. However, sporadic AD, the most common type of the disease, has not been associated with specific gene mutations suggesting genetic models do not faithfully represent all aspects of AD (Kawabata et al., 1991; Quon et al., 1991; Duff and Hardy, 1995). Hence, there is need for alternative animal models of AD.

Epidemiological studies have suggested a possible relationship between aluminum content in drinking water and AD (McLachlan et al., 1996; Altmann et al., 1999; Gauthier et al., 2000; Flaten, 2001; Exley and Esiri, 2006). Workers exposed in aluminum industry have shown impaired cognitive functions (Rifat et al., 1990; Polizzi et al., 2002; Giorgianni et al., 2003). The activities of antioxidant enzymes like superoxide dismutase, catalase, and glutathione peroxidase are reduced, while xanthine oxidase is enhanced following exposure to aluminum chloride (Shati et al., 2011). Exposure to aluminum chloride leads to accumulation of intermediary toxic compounds such as hydrogen peroxide and hydroxyl radicals, which may mediate the aluminum toxicity. Excessive intake of aluminum causes overexpression of amyloid precursor protein, deposition of amyloids in the central nervous system, and impairment in learning and memory in rats (Castorina et al., 2010). Aluminum has been shown to be colocalized with neurofibrillary tangles in AD patients. It induces degeneration of neurons in higher mammals (Crapper et al., 1973). Most importantly, chronic aluminum exposure in mice leads to pathology similar to that observed in AD patients (Luo et al., 2009). Although, several studies on aluminum chloride neurotoxicity are available, its impact on neurotransmission and neurometabolism has not been studied in detail.

Traditional Ayurveda claims to facilitate ‘healthy aging’ (Dwivedi et al., 2013), and thus has the possibility to alleviate the suffering from neurodegenerative disorders (Lakhotia, 2013). Rasa Sindoor (RS), an organo-metallic derivative of mercury and sulfur, is used as part of the rejuvenating Rasayana therapy, and is believed to promote long life with enhanced physical and mental strength (Sarkar and Chaudhary, 2010). RS was shown to improve general well-being of fruit flies. The dietary supplementation of RS prevented accumulation of inclusion bodies and heat shock proteins, improved the ubiquitin-proteasomal activity, suppressed apoptosis, elevated the levels of heterogeneous nuclear ribonucleoproteins and cyclic adenosine monophosphate response in Huntington and AD model in Drosophila (Dwivedi et al., 2013). Therefore, we hypothesized that RS intervention will improve memory and energy metabolism in AD mammalian model also.

Glutamate and γ-aminobutyric acid (GABA) are the major excitatory and inhibitory neurotransmitters, respectively, in the mature mammalian central nervous system. ^13^C-NMR measurements carried out *in vivo* with infusion of ^13^C-labeled substrates have established that neurotransmitters, glutamate, and GABA, energetics is supported by oxidative glucose metabolism in healthy brain (Sibson et al., 1998; Patel et al., 2004). In the current study, we employed ^1^H-[^13^C]-NMR spectroscopy in conjunction with infusion of [1,6-^13^C_2_]glucose in mice to investigate the energetics of glutamatergic and GABAergic neurotransmission in AlCl_3_ model of AD, and to evaluate the effects of RS intervention thereon. Our data suggest that exposure to AlCl_3_ adversely affected memory and metabolic activity of glutamatergic and GABAergic neurons in the hippocampus, cerebral cortex, and striatum. The RS intervention improved spatial memory and activity of excitatory and inhibitory neurons in AlCl_3_ treated mice.

## Materials and Methods

### Animal Preparation

All animal experiments were performed under approved protocol by Animal Ethics Committee of the Centre for Cellular and Molecular Biology (CCMB), Hyderabad, and were conducted in accordance with the guidelines established by Committee for the Purpose of Control and Supervision on Experiments on Animals, Ministry of Environment and Forests, Government of India. Two-month-old C57BL/6J male mice were housed in the CCMB Animal House in a room maintained at ∼22-25°C with relative humidity of 55–65% and 12 h/12 h light/dark cycle, which switches ON and OFF at 6:00 am and 6:00 pm, respectively.

### Administration of Aluminum Chloride and Rasa Sindoor (RS)

**FIGURE 1:**
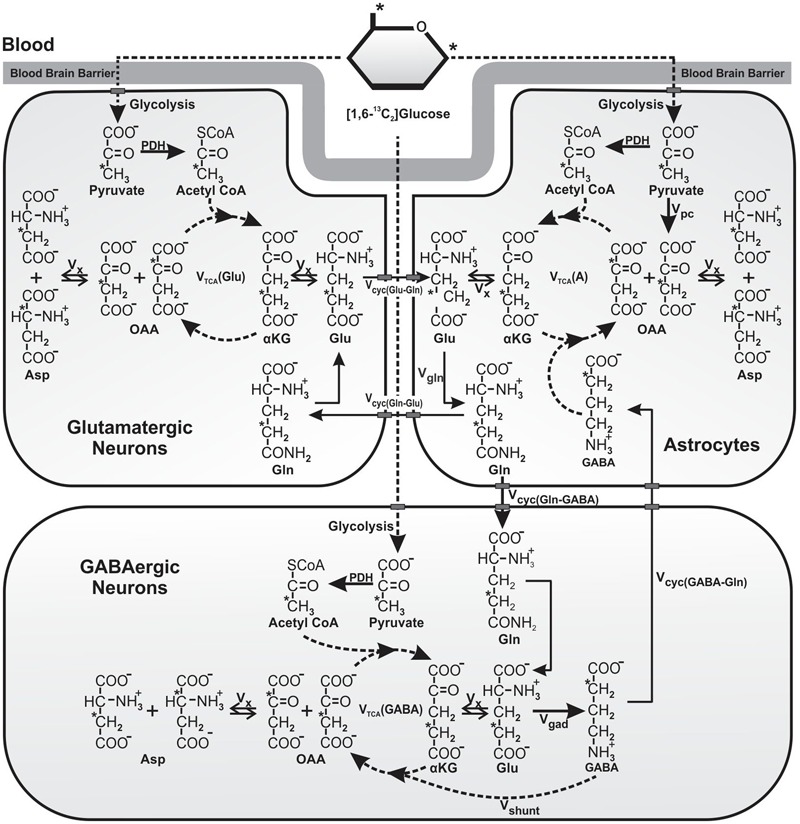
Schematic of ^13^C labeling of amino acids from [1,6-^13^C_2_]glucose. Metabolism of [1,6-^13^C_2_]glucose via glutamatergic and GABAergic TCA cycles labels [4-^13^C]glutamate, which is decarboxylated to [2-^13^C]GABA by GAD enzyme present specifically in GABAergic neurons. Labeling of [4-^13^C]glutamine occurs from [4-^13^C]glutamate and [2-^13^C]GABA via glutamate–glutamine and GABA–glutamine cycle. Further metabolism of [4-^13^C]glutamate and [2-^13^C]GABA in the corresponding TCA cycle incorporates label into [2-^13^C]/[3-^13^C]glutamate and [3-^13^C]/[4-^13^C]GABA, respectively. [3-^13^C]Pyruvate, the glycolytic product of [1,6-^13^C_2_]glucose is also metabolized by pyruvate carboxylase pathway (PC), and incorporates label into [2-^13^C]glutamine, [2-^13^C]glutamate, and [4-^13^C]GABA. For the simplicity, ^13^C labeling of amino acids from [1,6-^13^C_2_]glucose via the first turn of TCA cycle is depicted. Abbreviations used are: αKG, α-ketoglutarate; OAA, oxaloacetate; Asp, aspartate; GABA, γ-aminobutyric acid; Glu, glutamate; Gln, glutamine; V_cyc(GABA-Gln)_, GABA–glutamine cycling flux; V_cyc(Glu-Gln)_, glutamate–glutamine cycling flux; V_gad_, glutamate decarboxylase flux; V_gln_, glutamine synthesis rate; V_pc_, pyruvate carboxylase flux; V_shunt_, flux of GABA shunt; V_tca(A)_, Astroglial TCA cycle flux; V_tca(GABA)_, GABAergic TCA cycle flux; V_tca(Glu)_, glutamatergic TCA cycle flux; V_x_, exchange rate between α-ketoglutarate and glutamate. ^∗^Represents the position of ^13^C carbon.

**FIGURE 2:**
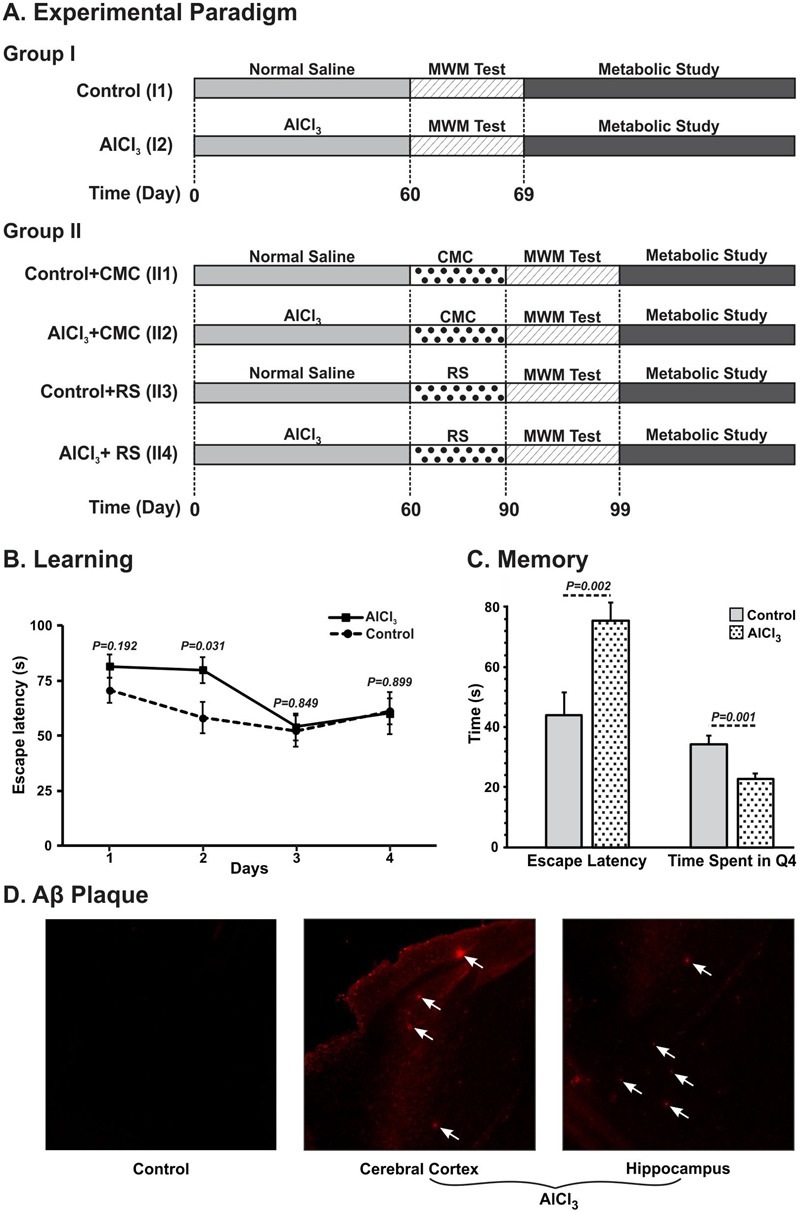
Schematic depiction of timeline of different treatments and measurements. **(AI)** Mice were administered AlCl_3_ (40 mg/kg, intraperitoneal) or normal saline once a day for 60 days. Mice were trained (61–64 day) and memory analysis (67^th^ day) was carried out using Morris Water Maze (MWM). Metabolic analysis was carried out on 69^th^ day by infusing [1,6-^13^C_2_]glucose, and measuring the labeling of amino acids in tissue extracts using ^1^H-[^13^C]-NMR spectroscopy. **(AII)** Mice were administered Rasa Sindoor (2 g/kg, intragastric) or CMC for 61 to 90 days following 60 days treatments of AlCl_3_ (40 mg/kg, intragastric) or normal saline. **(B)** Learning, and **(C)** Memory of mice following 60 days treatment of AlCl_3_ (40 mg/kg, intraperitoneal). Memory was assessed using Morris Water Maize test. **(D)** Immuno-histological depiction of Aβ plaques in AlCl_3_ treated mice. The arrows point to Aβ plaques in the AlCl_3_ treated mice.

Chronic exposure of AlCl_3_ in 2-month-old mice has been used to mimic the AD pathology in rodents (Xiao et al., 2011; Di Paolo et al., 2014). Mice were divided into two major sets of study. In the first set of study, animals were divided into two groups: **Ia**. Control (*n* = 12) and **Ib**. AlCl_3_ treated mice (*n* = 12) to assess the effects of Al exposure on memory and brain energy metabolism (**Figure 1**). Mice in Group **Ib** received AlCl_3_ (40 mg/kg) intraperitoneally once a day for 60 days, while Group **Ia** received normal saline (0.9% NaCl) for the same period (**Figure 2A** Group **I**). Aluminum chloride was purchased from Acros Organics. AlCl_3_ dissolved in normal saline (4 mg/ml) was administered (250 μl) to a 25-g mouse (40 mg/kg) between 1:00 and 2:00 pm once a day for 60 days.

In the second set of experiment, mice were divided into four subgroups: **IIa**. Control + Carboxy methyl cellulose, CMC (*n* = 7); **IIb**. AlCl_3_ + CMC (*n* = 6); **IIc**. Control + RS (*n* = 5); and **IId**. AlCl_3_ + RS (*n* = 5) to evaluate the effects of RS on memory and brain energy metabolism in AlCl_3_ exposed mice (**Figure 2A** Group **II**). Mice in Groups **IIb** and **IId** received AlCl_3_ (40 mg/kg, intragastrically) for 60 days, while those in **IIa** and **IIc** received normal saline for the same period. Following the 60 days of AlCl_3_ treatment, the Groups **IIc** and **IId** mice were administered RS in CMC (2 g/kg, intragastric) for 30 days, while Groups **IIa** and **IIb** received CMC. RS was procured from Arya Vaidya Sala, Kottakal, India. As per Ayurvedic Pharmacopeia of India, the shelf life of RS is infinite, since the HgS (Cinnabar) is known to be highly stable. For the present set of studies, we prepared a 20% suspension of RS in 1% CMC, and used within 2 days. All treatments of AlCl_3_ and RS in mice were carried out between 1:00 and 2:00 pm for the entire period of study.

### Memory Assessment

After 60 days of the AlCl_3_ treatment, memory of the mice was assessed using Morris Water Maze (MWM) test (Vorhees and Williams, 2006). The MWM consists of a circular pool with diameter and height, 120 and 47 cm, respectively, which was virtually divided into four equal quadrants. Different clues were provided on the wall of pool for spatial map of the environment. The pool was filled with water to a depth of 20 cm, and the water temperature was maintained between 22 and 25°C. An escape platform with 10 cm in diameter was submerged 0.5 cm under water level in the fourth quadrant. Mice were given 4 days of training, with four trials per day to locate the platform. Each training session was initiated from different quadrants and lasted for maximum period of 90 s. The time taken by mice (latency) to find the hidden platform was monitored with the help of Ethovision 3.1 software. The memory of mice was assessed on 7^th^ day by monitoring the time required by animals to reach the platform. Retention of memory in mice was assessed on 8^th^ day by removing the platform, and monitoring the time spent by animals in the platform zone. The learning and memory were also assessed following 30 days of RS intervention.

### Infusion of [1,6-^13^C_2_]Glucose

Overnight fasted mice were anesthetized with urethane (1.5 g/kg, intraperitoneal). The body temperature of the animals was maintained at ∼37°C. The respiration rate of the animals was monitored using BIOPAC device (Santa Barbara, CA, United States). The tail vein was cannulated for infusion of ^13^C labeled substrate. [1,6-^13^C_2_]Glucose was purchased from ISOTEC, Miamiburg, United States, and dissolved (0.225 mol/L) in deionized water for infusion. [1,6-^13^C_2_]Glucose was administered as a bolus (1013 μmol/kg in 15 s), followed by an exponentially decreasing infusion rate every 0.5 min for the next 8 min, whereupon the rate was maintained to 51 μmol/kg/min until *in situ* freezing of the brain (Fitzpatrick et al., 1990; Patel et al., 2005; Tiwari et al., 2013). Mice in Group I were administered [1,6-^13^C_2_]glucose for 10, 30, 60, and 90 min (*n* = 3 at each time point) to derive the ^13^C labeling kinetics of amino acids for metabolic analysis. Mice (*n* = 23) in second set of study were administered [1,6-^13^C_2_]glucose for 10 min (one time point) for estimation of metabolic flux using the initial rate approximation. Blood was collected from orbital sinus just before the end of the infusion, and the brain was frozen *in situ* in *liq.*N_2_ and stored at -80°C till further processing.

### Extraction of Neurometabolites from Brain Tissue

Brains were chiseled out and dissected into different regions such as cerebral cortex, hippocampus, and striatum under frozen conditions in a cryostat maintained at -20°C. Metabolites were extracted from frozen brain tissues (Patel et al., 2001). In brief, brain tissues were powdered with 0.1 mol/L HCl in methanol (1:2 w/v) using glass homogenizer maintained in a dry-ice-ethanol bath. [2-^13^C]Glycine (0.2 μmol, 99 atom%, Cambridge Isotopes, Andover, MA, United States) was added as a concentration reference. The powdered tissues were homogenized with ice-cold ethanol and centrifuged at 12,000 g for 30 min at 4°C to remove cell debris. The dried powder was dissolved in phosphate buffered deuterium oxide containing sodium trimethylsilylpropionate (0.25 mmol/L) for NMR analysis.

### Analysis of Plasma Glucose

Blood plasma (100 μl) obtained from orbital sinus was mixed with deuterium oxide (450 μl) containing sodium formate (1 mmol/L), and passed through a centrifugal filter (10-kDa cutoff, VWR, Radnor, PA, United States) to remove macromolecules (Tiwari et al., 2013). Isotopic ^13^C labeling of glucose was determined in the plasma using ^1^H NMR spectroscopy at 600 MHz spectrometer. The concentration of glucose was determined from the area of glucose resonance (5.2 ppm) relative to formate. The percent ^13^C enrichment of glucose-C1 was calculated by dividing the area of the ^13^C-coupled satellites by the total ^1^H area (^12^C+^13^C) measured at 5.2 ppm.

### NMR Spectroscopy of Brain Extracts

^1^H-[^13^C]-NMR spectra of brain tissue extracts were recorded at 600 MHz NMR spectrometer (Bruker AVANCE II, Karlsruhe, Germany) using a triple resonance probe equipped with three axes gradients (de Graaf et al., 2003). NMR parameters used are as described in detail in Bagga et al. (2013). In brief, a typical ^1^H-[^13^C]-NMR experiment involved collection of two spin-echo ^1^H NMR spectra under the condition of an OFF/ON ^13^C inversion pulse. Free induction decays (FIDs) were zero filled, apodized to Lorentzian line broadening, Fourier transformed, and phase corrected. The C-13 edited NMR spectrum was obtained by subtracting the spectrum obtained with ^13^C inversion pulse from that acquired under without inversion pulse. The concentrations of metabolites were calculated from the peak area in the spectrum obtained without ^13^C inversion pulse (OFF) relative to [2-^13^C]glycine. The isotopic ^13^C enrichments of amino acids at different carbon positions were determined from the ratio of area in the difference spectrum and the non-edited spectrum.

### Estimation of Aluminum and Mercury in Brain

The aluminum and mercury content in the cerebral cortex of the Group II mice was measured using inductively coupled plasma mass spectrometry (ICP-MS). In brief, the weighed amount of cerebral cortex was digested using plastic pestle in concentrated nitric acid (1 ml). The resulting homogenate was diluted to 10 ml, and analyzed by ICP-MS for aluminum and mercury content.

### Immuno-Histochemical Analysis of Aβ Plaques in Brain

The Aβ plaques were visualized by immuno-staining in first set of experiment following 60 days of AlCl_3_ treatment. Bain samples were washed with phosphate buffered saline (PBS) followed by permeabilization with 0.1% Tween 20. The axial slices (30 μm) of brain were made using cryo-microtome, and stored in PBS with 0.1% sodium azide. For immuno-staining, sections were washed with PBS, blocked with 5% serum in PBS with 0.1% Tween 20, followed by overnight incubation at 4°C with primary Aβ antibody against the first 14 amino acids of the Aβ peptide (Abcam ab2539). The sections were washed thrice with PBS and treated with appropriate secondary antibody at 4°C for 1 h. The slides were washed again, mounted using Vectashield, and examined Confocal microscope (Leica SP5 AOBS) with a 10x dry objective.

### Determination of Metabolic Rates from Kinetic Data

A three-compartment metabolic model (Patel et al., 2005; Tiwari et al., 2013) was fitted to the time courses of ^13^C labeling of amino acids from [1,6-^13^C_2_]glucose in the first set of study using a CWave software package (Mason et al., 2003) to determine the metabolic fluxes (**Figure 1**). The metabolic model consists of a series of differential equations describing mass balance and ^13^C isotope flowing from [1,6-^13^C_2_]glucose (Supplementary Table 1). The ^13^C turnover curves of [3-^13^C]glutamate, [4-^13^C]glutamate, [2-^13^C]GABA, [3-^13^C]GABA, [3-^13^C]aspartate and [4-^13^C]glutamine were used for the analysis. The averaged concentrations of glutamate, glutamine, GABA and aspartate measured (Supplementary Table 2) in tissue extracts were used for metabolic modeling. Glutamine and GABA were assumed to be localized in astroglia and GABAergic neurons, respectively, whereas glutamate pool was distributed differently in brain regions as determined previously (Supplementary Table 1). The ratio V_cyc(Glu-Gln)_/V_tca(Glu)_ and V_cyc(GABA-Gln)_/V_tca(GABA)_ were constrained to values for the cerebral cortex, hippocampus and striatum as presented in Supplementary Table 1 (Tiwari et al., 2013). The values of V_dil(Gln)_, V_pc_, V_tca(ANet)_, V_dil(A)_, V_tca(GABANet)_, V_shunt_, V_dil(GABA)_, V_tca(Glu)_, V_dil(Glu)_ and V_xGlu(Glu-KG)_ were varied during modeling. The uncertainty in estimation of these parameters was determined using a Monte Carlo analysis as described previously by Mason et al. (1992). Briefly, 500 noisy data sets were generated by adding the random Gaussian noise to the noiseless set of data. The model was fitted to the data to generate a list of 500 values for each of the rates. These values were used to calculate average and standard deviation of each fitted parameters.

### Estimation of Glucose Oxidation by Neurons

The rates of neuronal glucose oxidation in the second set of study were calculated based on the labeling of amino acids from 10-min infusion of [1,6-^13^C_2_]glucose as described earlier (Patel et al., 2005; Bagga et al., 2013). The apparent cerebral metabolic rate of glucose oxidation (CMR_Glc(ox)_) by glutamatergic neurons was obtained as follows:

(1) $${\text{CMR}}_{\text{Glc}(\text{Glu})}\text{= 0}.\text{5x}(\frac{\text{1}}{\text{10}})\text{x}(\frac{\text{1}}{{\text{Glc}}_{\text{1}}})\text{x}\{\text{0}.\text{82}[\text{Glu}]x({\text{Glu}}_{\text{4}}{\text{+2Glu}}_{\text{3}})\text{+0}.\text{42}[\text{Asp}]({\text{2Asp}}_{\text{3}})\}$$

where Glu_i_ and Asp_i_ are percentage ^13^C labeling of glutamate and aspartate, respectively, at carbon ‘*i’* from [1,6-^13^C_2_]glucose during 10-min infusion, Glc_1_ is the labeling of [1-^13^C]glucose in blood plasma, and [Glu] and [Asp] are the concentrations of glutamate and aspartate, respectively.

The cerebral metabolic rate of glucose oxidation by GABAergic neurons was estimated as follows:

(2) $$\begin{array}{c} {\text{CMR}}_{\text{Glc}(\text{GABA})}\text{=0}.\text{5}x(\frac{\text{1}}{\text{10}})x(\frac{\text{1}}{{\text{Glc}}_{\text{1}}})x\;\{\text{0}.\text{02}[\text{Glu}]({\text{Glu}}_{\text{4}}{\text{+2Glu}}_{\text{3}})\\ \text{ +}[\text{GABA}]({\text{GABA}}_{\text{2}}{\text{+2GABA}}_{\text{4}})\text{+0}.\text{42}[\text{Asp}]({\text{2Asp}}_{\text{3}})\} \end{array}$$

GABA_i_ is the percentage labeling of GABA at carbon *‘i’* from [1,6-^13^C_2_]glucose, and [GABA] is the concentration of GABA in the cerebral cortex.

The total glucose oxidation was estimated as:

(3) $$\begin{array}{c} {\text{CMR}}_{\text{Glc}(\text{Total})}\text{=0}.\text{5}x(\frac{\text{1}}{\text{10}})x(\frac{\text{1}}{{\text{Glc}}_{\text{1}}})x\;\{[\text{Glu}]({\text{Glu}}_{\text{4}}{\text{+2Glu}}_{\text{3}})\text{+}\\ \left[\text{GABA}]({\text{GABA}}_{\text{2}}{\text{+2GABA}}_{\text{4}})\text{+}[\text{Asp}]({\text{2Asp}}_{\text{3}})\text{+}[\text{Gln}]({\text{Gln}}_{\text{4}})\right\} \end{array}$$

### Statistics

The Student’s *t*-test was applied to determine the significance of difference in the memory, concentration and metabolic rates in AlCl_3_ treated mice when compared with controls in the first set of study. One-way ANOVA was carried to determine the statistical significance of difference in the concentration, ^13^C labeling, and cerebral metabolic rate of glucose oxidation with RS intervention in the second set of study. The *post hoc* Tukey’s honest test was carried out to further identify the statistical significance of difference among groups. All results are reported as mean ± standard error of the mean (SEM).

## Results

### Effects of AlCl_3_ on Learning and Memory of Mice

The learning and memory of AlCl_3_ treated mice were assessed through the MWM test after 60 days of treatment and compared with parallel saline treated control mice. The escape latency, the time to locate the platform, is significantly longer (*p* = 0.031) on the 2^nd^ day of training in AlCl_3_ treated mice when compared with controls (**Figure 2B**). Additionally, AlCl_3_ treated mice (75.4 ± 6.1 s, *n* = 12) took significantly (*p* < 0.002) longer time during memory test to reach the hidden platform when compared with normal saline treated controls (43.9 ± 7.8 s, *n* = 12) (**Figure 2C**). Moreover, these mice spent (22.8 ± 1.6 s, *n* = 12) significantly (*p* < 0.001) less time in the platform zone than controls (34.4 ± 1.6 s, *n* = 12). These data suggest that AlCl_3_ exposure leads to impairment in the memory in mice.

### Aluminum Content in Cortical Tissue

**FIGURE 3:**
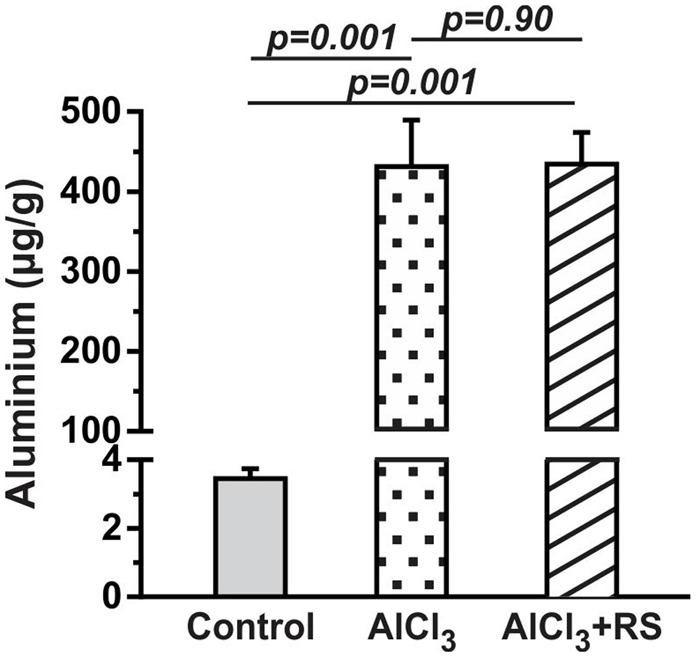
The level of aluminum in the cerebral cortex of mice after 2 months AlCl_3_ treatment. The aluminum concentration was measured in brain homogenate using inductively coupled plasma mass spectrometry.

The level of aluminum in the cerebral cortex of control mice was found to be 3.4 ± 0.7 μg per gram of brain tissue (*n* = 5). The treatment of AlCl_3_ in mice for 2 months led to significant (*p* = 0.002) increase in aluminum content in AlCl_3_ treated animals (431.4 ± 129.3 μg per g of brain tissue, *n* = 5) (**Figure 3**). There was no significant (*p* = 0.90) difference for the aluminum content between AlCl_3_+RS and AlCl_3_+CMC group.

### Effects of AlCl_3_ on Neurometabolites Homeostasis

**FIGURE 4:**
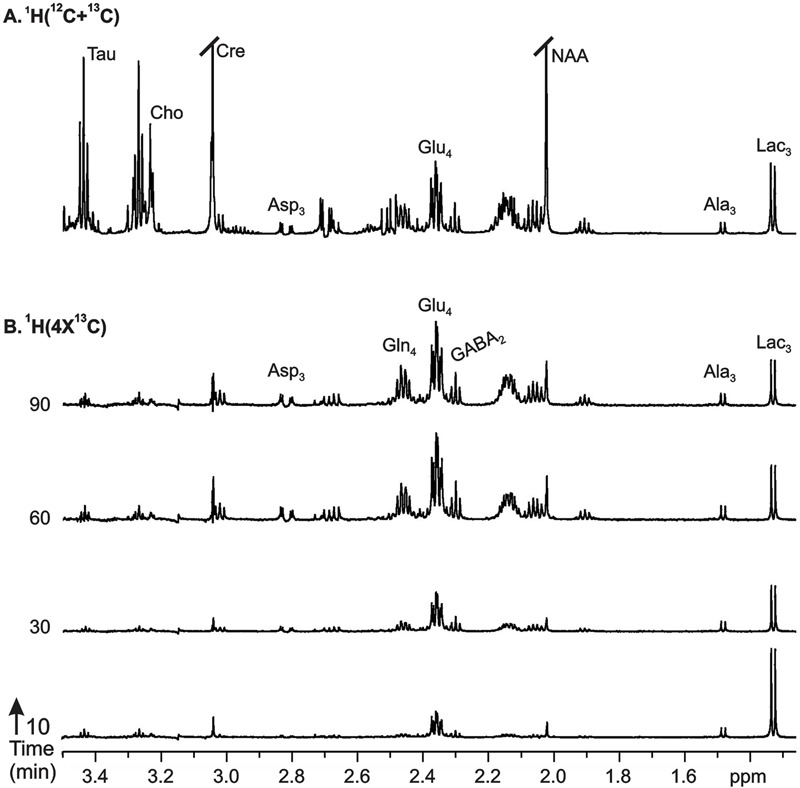
Representative ^1^H-[^13^C]-NMR spectra from cortical tissue extracts of AlCl_3_ treated mice: **(A)**^1^H-[^12^C+^13^C] represents total concentration of metabolites, **(B)**^1^H-[4x^13^C] depicts ^13^C labeling of metabolites from [1,6-^13^C_2_]glucose. Mice were infused with [1,6-^13^C_2_]glucose for pre-defined time, and ^1^H-[^13^C]-NMR spectra were recorded from cortical tissue extracts. Peak labels are: Ala_C3_, alanine-C3; Asp_C3_, asparate-C3; GABA_C2_, GABA-C2; Gln_C4_, glutamine-C4; Glu_C4_, glutamate-C4.

The levels of neurometabolites were measured in non-edited ^1^H-[^13^C]-NMR spectrum of brain tissue extracts relative to [2-^13^C]glycine (**Figure 4A**). The levels of cortical neurometabolites remain unchanged (*p* > 0.2) following 60 days exposure of AlCl_3_ (Supplementary Table 2). The levels of glutamate and N-acetyl aspartate (NAA) were significantly (*p* < 0.01) lower in the striatum in AlCl_3_ treated mice (*n* = 12) than in normal saline treated controls (*n* = 12). Moreover, a significant (*p* < 0.001) increase in myo-inositol level was observed in hippocampus of the AlCl_3_ treated mice when compared with NS treated controls.

### Effects of AlCl_3_ on ^13^C Labeling of Brain Amino Acids from [1,6-^13^C_2_]Glucose

The cerebral metabolism was followed by ^1^H-[^13^C]-NMR spectroscopy in brain tissue extracts in conjunction with timed infusion of [1,6-^13^C_2_]glucose. The percent ^13^C labeling of plasma [1-^13^C]glucose averaged during entire period of infusion was found to be similar in AlCl_3_ treated mice (44.9 ± 5.7%, *n* = 12) and normal saline treated controls (40.8 ± 4.3%, *n* = 12), suggesting that AlCl_3_ did not perturb homeostasis of plasma glucose.

The ^13^C labeling of cortical amino acids was measured *ex vivo* in brain tissue extracts using ^1^H-[^13^C]-NMR spectroscopy. The ^13^C labeling of [4-^13^C]glutamate, [3-^13^C]alanine, and [3-^13^C]lactate was visible at 10-min infusion time in AlCl_3_ treated mice (**Figure 4B**). The labeling of different carbons of GABA, glutamine, and aspartate, which were very small at 10 min, could be seen from 30 min onward, and reached to steady state level by 60 min. A similar labeling pattern was observed in control mice. The percent ^13^C labeling of cortical metabolites at different carbon positions was calculated, and plotted with time to construct the ^13^C turnover curve of different amino acids from [1,6-^13^C_2_]glucose. A similar analysis was performed to generate the ^13^C turnover curves of amino acids in striatum and hippocampus.

### Effects of AlCl_3_ Exposure on Metabolic Rates

**FIGURE 5:**
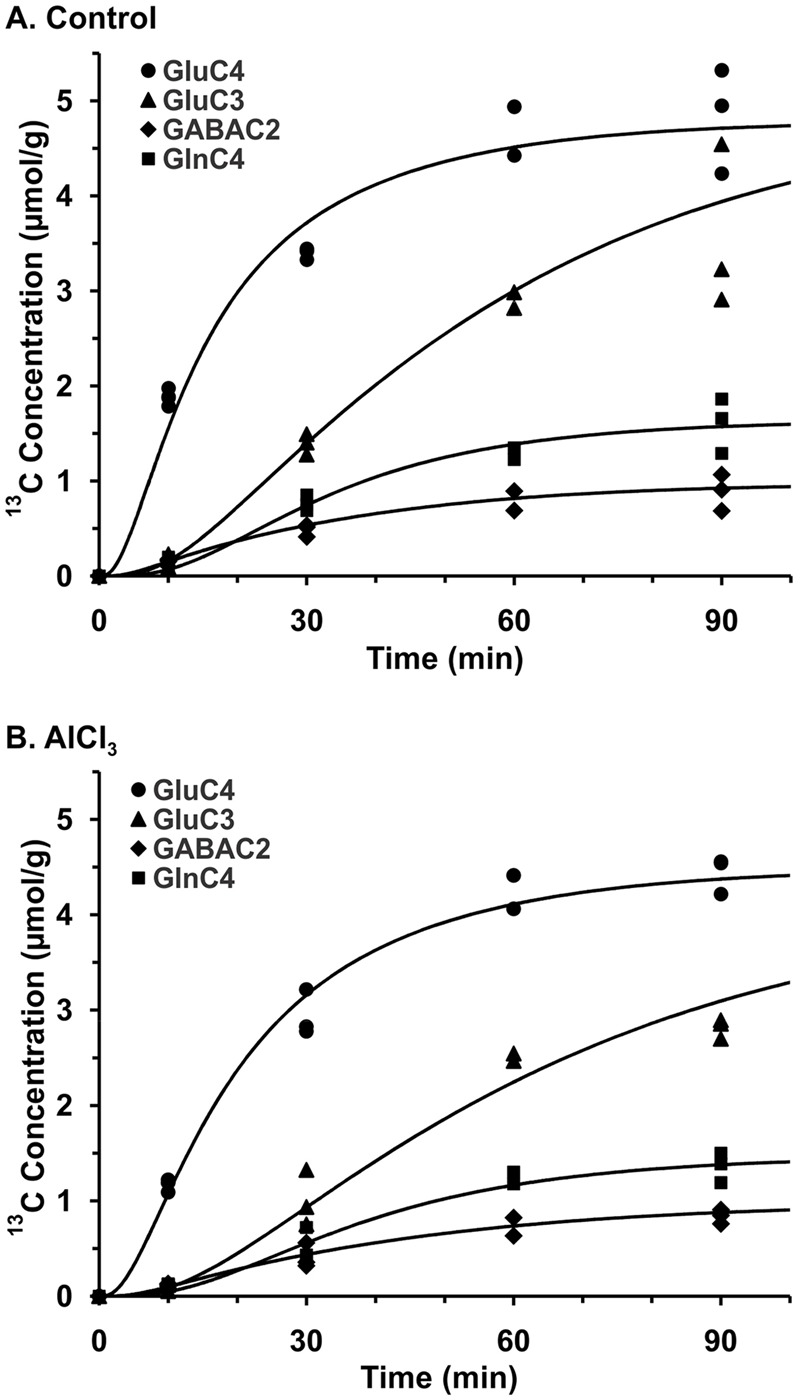
Fit of three compartment metabolic model to ^13^C turnover of cortical amino acids in **(A)** Control, **(B)** AlCl_3_ treated mice. Mice were infused with [1,6-^13^C_2_]glucose for pre-defined time. ^13^C Concentrations of cortical amino acids were measured in extracts in ^1^H-[^13^C]-NMR spectra. Symbols represent the measured ^13^C labeling, while lines show the best fit of the three compartment metabolic model.

The metabolic rates associated with glutamatergic and GABAergic pathways were obtained by modeling of ^13^C labeling of amino acids from glucose. The best fit of the metabolic model (Supplementary Table 1) to the ^13^C turnover of amino acids in the cerebral cortex is presented in **Figure 5**. Each symbol represents ^13^C labeling measured from individual animals (*n* = 12), while the line depicts the prediction of labeling based on metabolic model and optimum values of different iterated parameters. The quality of fit of the model to the ^13^C turnover of amino acids in the hippocampus and striatum was similar to that in the cerebral cortex.

#### Cerebral Cortex

**FIGURE 6:**
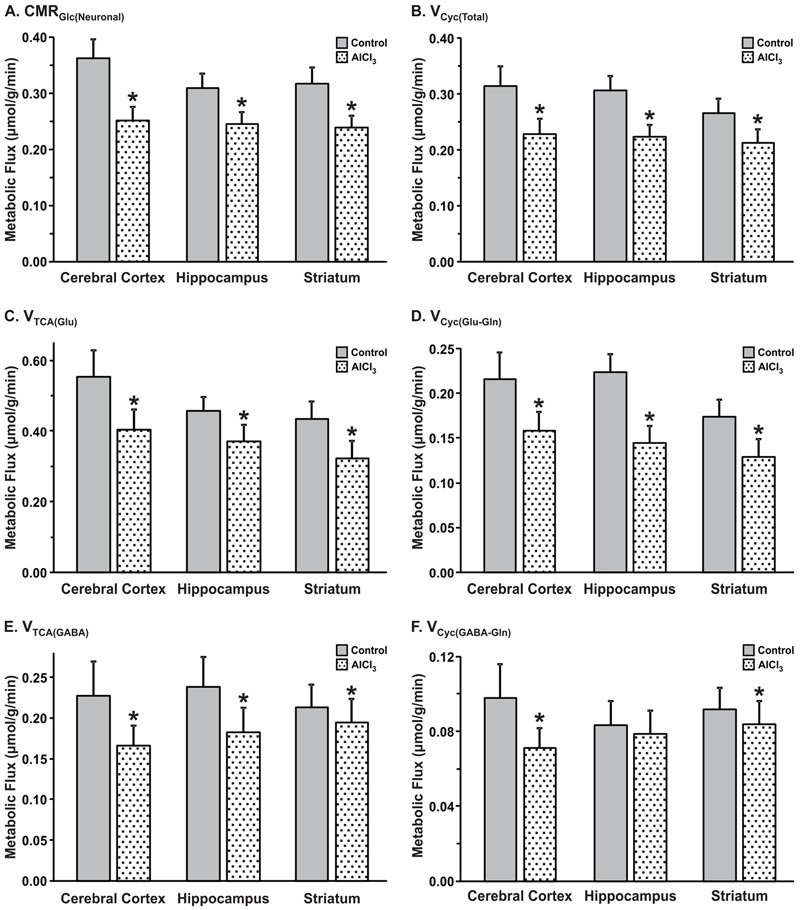
Effects of AlCl_3_ treatment on **(A)** Neuronal metabolic rate of glucose oxidation, **(B)** Neurotransmitter cycling rates. **(C,E)** tricarboxylic acid cycle flux; **(D,F)** neurotransmitter cycling flux of glutamatergic **(C,D)** and GABAergic neurons **(E,F)**. Metabolic fluxes were obtained by analysis of ^13^C turnover of amino acids from [1,6-^13^C_2_]glucose using a three compartment metabolic model. The error in the rates was determined by performing the Monte-Carlo analysis. Values represent mean ± SEM. ^∗^*p* < 0.001.

AlCl_3_ exposure considerably perturbed the metabolic activity in the cerebral cortex. The rate of neuronal glucose oxidation was found to be decreased by 30% in mice exposed to AlCl_3_ (0.252 ± 0.024 μmol/g/min) as compared with normal saline treated controls (0.362 ± 0.034 μmol/g/min; **Figure 6A**). The neurotransmitter cycling flux was also decreased to similar extent (Controls: 0.314 ± 0.035 μmol/g/min; AlCl_3_: 0.229 ± 0.026 μmol/g/min, *p* < 0.001) with AlCl_3_ exposure (**Figure 6B**). Further analysis indicated that the reduction in TCA cycle flux of glutamatergic (0.405 ± 0.055 vs. 0.554 ± 0.076 μmol/g/min, *p* < 0.001) and GABAergic neurons (0.166 ± 0.025 vs. 0.228 ± 0.042 μmol/g/min, *p* < 0.001) together have contributed to metabolic deficit in AlCl_3_ treated mice (**Figures 6C,E**). Moreover, the rates of glutamate–glutamine and GABA–glutamine cycle were decreased with AlCl_3_ exposure (**Figures 6D,F**).

#### Hippocampus

The impact of AlCl_3_ exposure in mice resulted in relatively smaller decrease in hippocampal metabolic rate as compared with that in the cerebral cortex (**Figures 6A,B**). The glutamatergic TCA cycle and glutamate–glutamine cycle fluxes were decreased in mice treated with AlCl_3_ (**Figures 6C,D**). The GABAergic TCA cycle (0.190 ± 0.031 vs. 0.239 ± 0.037 μmol/g/min, *p* < 0.001) was found to be decreased significantly following AlCl_3_ exposure (**Figures 6E,F**).

#### Striatum

The impact of AlCl_3_ exposure on striatal metabolic activity was in between the hippocampus and cerebral cortex. The rates of neuronal glucose oxidation (0.224 ± 0.019 vs. 0.285 ± 0.029 μmol/g/min, *p* < 0.001) and neurotransmitter cycle (0.158 ± 0.019 vs. 0.183 ± 0.022 μmol/g/min, *p* < 0.001) were reduced significantly with AlCl_3_ treatment (**Figures 6A,B**). The reduction in neuronal glucose oxidation and neurotransmitter cycle rates in striatum was mostly contributed by corresponding decrease in the glutamatergic TCA cycle (0.308 ± 0.047 vs. 0.379 ± 0.049 μmol/g/min, *p* < 0.001) and glutamate–glutamine cycle (0.074 ± 0.011 vs. 0.091 ± 0.012 μmol/g/min, *p* < 0.001). The relative changes in rates of GABAergic TCA cycle and neurotransmitter cycle were smaller in the striatum following AlCl_3_ exposure (**Figures 6E,F**).

These data suggest that exposure of AlCl_3_ for a period of two months has differential effect on glutamatergic and GABAergic metabolic activity across brain.

### Cerebral Metabolism after One Month of AlCl_3_ Exposure

The metabolic rate of glucose oxidation associated with different cell types in the cerebral cortex was reduced following AlCl_3_ exposure in Group II mice (**Figures 8B,C**). It is worth noting that the magnitude of reduction in CMR_Glc(ox)_ was higher (>32%) in the hippocampal and striatal regions following a waiting of one month as compared with that measured immediately in first set of study. The impact of one month waiting period was relatively more on the metabolic activity of GABAergic neurons in the striatum, which reduced by 28% as compared with only 8% reduction immediately after AlCl_3_ treatment. The glutamatergic rate that was decreased by 19% immediately after AlCl_3_ exposure was found to be reduced by 33% after one month of waiting period. The most dramatic effects of one month waiting period was observed in hippocampal region, wherein metabolic deficit was found in the range of 34–49% (Group II mice), while it was only 14–20% in the first set of experiment measured immediately after AlCl_3_ exposure.

### RS Intervention Improves Memory in AlCl_3_ Treated Mice

**FIGURE 7:**
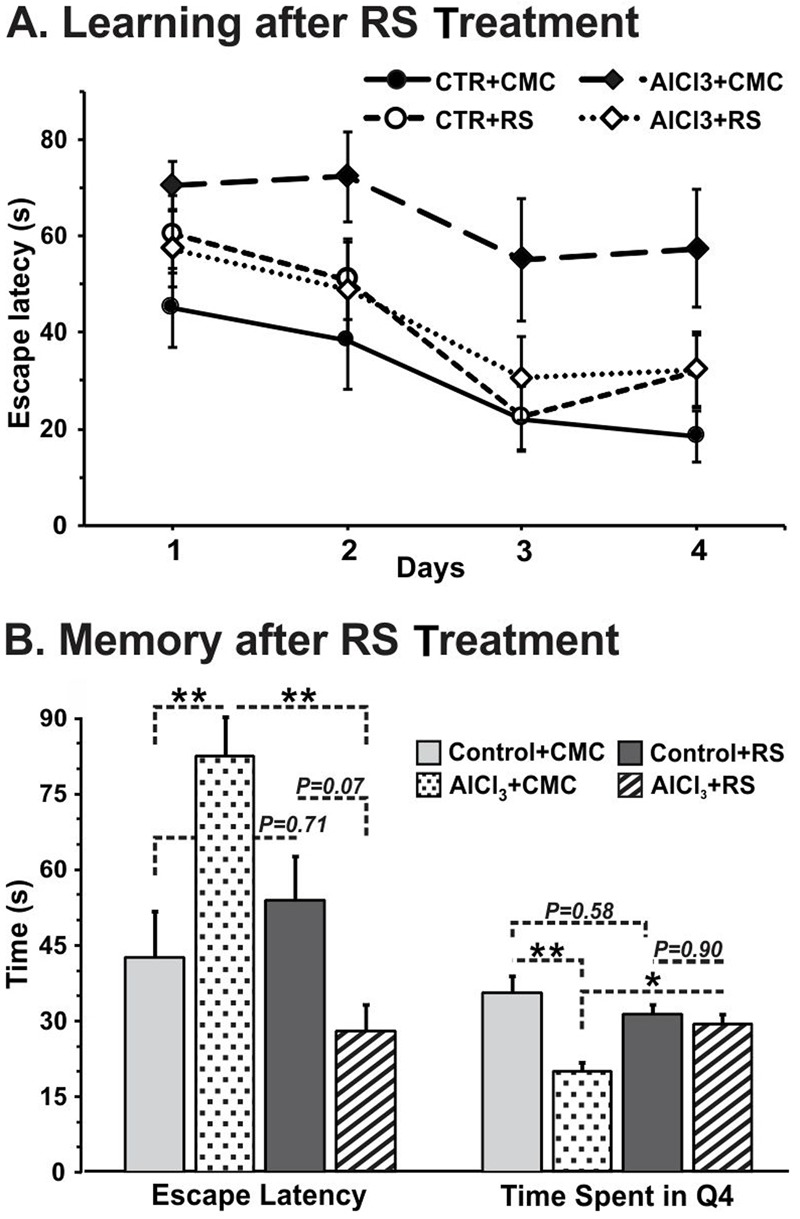
**(A)** Learning, and **(B)** Memory of mice following Rasa Sindoor (RS) intervention. Mice were administered RS (2 g/kg, intragastric) for 61–90 days following 60 days treatments of AlCl_3_ (40 mg/kg, intragastric). The memory was assessed using Morris Water Maze. The statistical analysis suggested no significant (*F*[3,32] ≤ 2.359, *p* ≥ 0.09) difference in learning among different groups of mice.

A test for the retention of memory without platform indicated that the time spent around the platform area is significantly (*p* = 0.001) less in AlCl_3_ exposed mice. Intervention of RS to AlCl_3_ treated mice for one month period was found to decrease the escape latency (28.1 ± 5.1 s, *n* = 7) significantly (*p* = 0.001*)* as compared with the vehicle (CMC) treated AlCl_3_ mice (82.6 ± 7.4 s, *n* = 5). Furthermore, analysis of retention of memory indicated that time spent around the platform zone is also increased significantly (*p* = 0.04) following RS administration (AlCl_3_+CMC 20.0 ± 1.9 s, *n* = 5; AlCl_3_+RS 29.7 ± 1.4 s, *n* = 6, **Figure 7B**). It is noteworthy that RS intervention in control mice had no significant effect on the escape latency (*p* = 0.71) (**Figure 7A**) or on the time spent around the platform (*p* = 0.58). Although the average latency to reach the platform appears to be higher in Control+RS mice than AlCl_3_+RS group, the difference is not significant (*p* = 0.069). Moreover, the latency of Control+RS mice (54.0 ± 8.6 s) was not significantly (*p* = 0.71) different from Control+CMC (42.7 ± 9.0 s).

There was no significant (*p* = 0.90) difference for the aluminum content between AlCl_3_+RS and AlCl_3_+CMC group (**Figure 3**). Moreover, the analysis of mercury indicated no significant increase in mercury level in RS treated mice (Supplementary Figure 1). These data suggest that RS intervention has improved memory in AlCl_3_ exposed mice without any adverse effect on memory in controls.

### RS Intervention Improved Brain Energy Metabolism in AlCl_3_ Treated Mice

As mentioned above, the ^13^C labeling of cortical [4-^13^C]glutamate (*p* = 0.049), [3-^13^C]glutamate (*p* = 0.001), [3-^13^C]aspartate (*p* = 0.001), and [4-^13^C]glutamine (*p* = 0.009) was found to be reduced significantly in AlCl_3_ treated mice as compared with the normal saline treated controls (**Table 1**). The RS intervention in AlCl_3_ exposed mice increased ^13^C labeling of cortical [4-^13^C]glutamate (AlCl_3_+CMC 1.61 ± 0.05, *n* = 5; AlCl_3_+RS 1.91 ± 0.05 μmol/g, *n* = 7, *p* = 0.057) and [3-^13^C]aspartate (AlCl_3_+CMC 0.12 ± 0.01, *n* = 5; AlCl_3_+RS 0.17 ± 0.01 μmol/g, *n* = 7, *p* = 0.034) when compared with CMC treated mice (**Table 1** and Supplementary Figure 2). Though, the ^13^C labeling of [4-^13^C]glutamine was increased with RS supplementation in AlCl_3_ treated mice (AlCl_3_+CMC 0.19 ± 0.01, *n* = 5; AlCl_3_+RS 0.25 ± 0.02, *n* = 7, μmol/g), it did not reach to statistical significance (*p* = 0.067). In addition to improvement in the ^13^C labeling of these amino acids, a robust increase in the labeling of [2-^13^C]GABA, [3-^13^C]aspartate, and [4-^13^C]glutamine in the subcortical regions, namely hippocampus and striatum, was observed (**Table 1**). It is worth noting that RS did not affect the ^13^C labeling of amino acids in control mice (*p* > 0.20).

**Table 1:** Concentration (μmol/g) of ^13^C labeled amino acids from [1,6-^13^C_2_]Glucose in RS-treated mice.

Brain region	Treatment group	Amino acids					
		Glu_C4_	GABA_C2_	Gln_C4_	Asp_C3_	Glu_C3_	GABA_C4_
Cerebral Cortex	Control+CMC	1.97 ± 0.09	0.21 ± 0.02	0.29 ± 0.02	0.20 ± 0.02	0.42 ± 0.07	0.08 ± 0.02
	Control+RS	1.99 ± 0.13	0.20 ± 0.01	0.25 ± 0.01	0.17 ± 0.01	0.32 ± 0.02	0.05 ± 0.01
	AlCl_3_+CMC	1.61 ± 0.06^∗^	0.16 ± 0.01	0.19 ± 0.01^∗∗^	0.12 ± 0.01^∗∗^	0.23 ± 0.02^∗∗^	0.04 ± 0.01
	AlCl_3_+RS	1.91 ± 0.05	0.21 ± 0.01	0.25 ± 0.02	0.17 ± 0.01^#^	0.29 ± 0.01	0.05 ± 0.01
Hippocampus	Control+CMC	1.67 ± 0.07	0.31 ± 0.02	0.25 ± 0.02	0.17 ± 0.01	0.34 ± 0.05	0.09 ± 0.01
	Control+RS	1.64 ± 0.08	0.29 ± 0.02	0.23 ± 0.01	0.14 ± 0.01	0.27 ± 0.02	0.08 ± 0.01
	AlCl_3_+CMC	1.24 ± 0.06^∗∗^	0.19 ± 0.01^∗∗^	0.16 ± 0.01^∗∗^	0.10 ± 0.01^∗∗^	0.17 ± 0.02^∗∗^	0.04 ± 0.01^∗∗^
	AlCl_3_+RS	1.61 ± 0.09^#^	0.27 ± 0.01^#^	0.24 ± 0.02^#^	0.16 ± 0.02^##^	0.28 ± 0.04^#^	0.08 ± 0.01^#^
Striatum	Control+CMC	1.34 ± 0.11	0.22 ± 0.02	0.18 ± 0.02	0.12 ± 0.01	0.30 ± 0.03	0.07 ± 0.02
	Control+RS	1.29 ± 0.06	0.19 ± 0.01	0.17 ± 0.01	0.12 ± 0.01	0.22 ± 0.02	0.05 ± 0.01
	AlCl_3_+CMC	0.86 ± 0.06^∗∗^	0.14 ± 0.01^∗∗^	0.11 ± 0.01^∗∗^	0.06 ± 0.01^∗^	0.11 ± 0.02^∗∗^	0.04 ± 0.01
	AlCl_3_+RS	1.20 ± 0.07^#^	0.18 ± 0.01	0.15 ± 0.01	0.11 ± 0.02	0.21 ± 0.02^#^	0.05 ± 0.01

*Mice were infused with [1,6-^*13*^C_*2*_]glucose for 10 min. The concentration of ^*13*^C labeled amino acids were measured in tissue extracts in ^*13*^C edited spectrum using [2-^*13*^C]glycine as standard. Values are presented as mean ± SEM. ^∗^*p* < 0.05, ^∗∗^*p* < 0.001 when AlCl_*3*_+CMC animals were compared with Control+CMC, and ^#^*p* < 0.01, ^##^*p* < 0.01 when AlCl_*3*_+RS mice were compared with AlCl_*3*_+CMC.*

**FIGURE 8:**
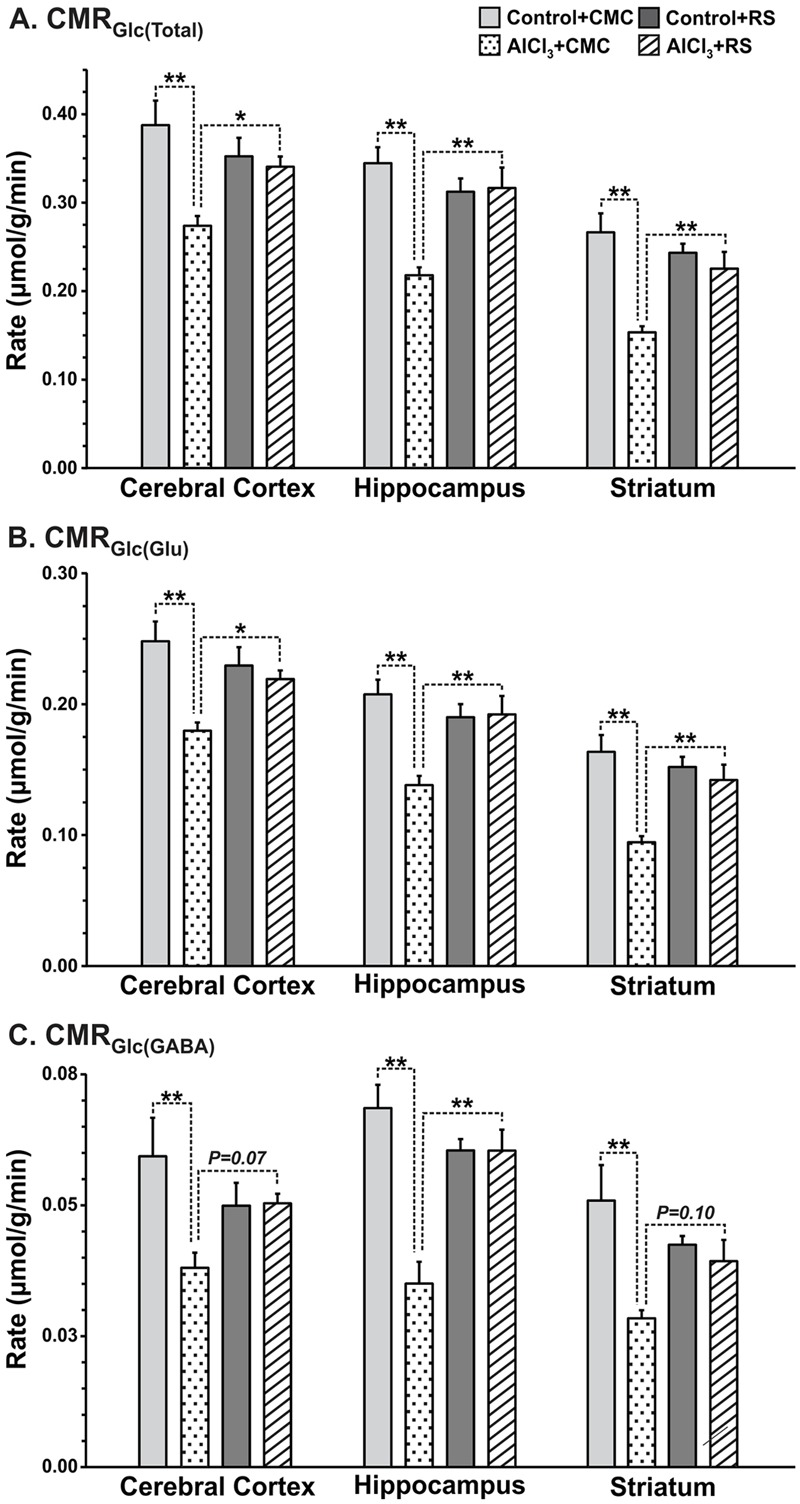
Effects of Rasa-sindoor intervention on cerebral metabolic rates of glucose oxidation (CMR_Glc(ox)_): **(A)** Total, **(B)** Glutamatergic Neurons, and **(C)** GABAergic neurons in the cerebral cortex, hippocampus and striatum in AlCl_3_ treated mice. Mice were infused with [1,6-^13^C_2_]glucose for 10 min. The CMR_Glc(ox)_ was calculated based on the ^13^C labeled trapped into different amino acids using Equations 1–3 as described in the Methods section. Values are presented as mean ± SEM. ^∗^*p* = 0.01, ^∗∗^*p* = 0.001. The number of mice used in different groups were: Control + CMC (*n* = 7); Control + RS (*n* = 5); AlCl_3_ + CMC (*n* = 6) and AlCl_3_ + RS (*n* = 5).

The cerebral metabolic rates of glucose oxidation in the second set of study, calculated using Equations 1–3, are presented in **Figure 8**. The RS intervention improved the rate of glucose oxidation in the cerebral cortex (AlCl_3_+CMC 0.270 ± 0.011, *n* = 5; AlCl_3_+RS 0.341 ± 0.011, μmol/g/min, *n* = 7, *p* = 0.028) in AlCl_3_ exposed mice. Analysis of energy metabolism at the cellular level revealed that recovery of CMR_Glc_ in glutamatergic neurons (AlCl_3_+CMC 0.180 ± 0.006, *n* = 5; AlCl_3_+RS 0.219 ± 0.007, μmol/g/min, *n* = 7, *p* = 0.044) was better than that in GABAergic neurons (*p* = 0.07). It is noteworthy that RS intervention did not affect (*p* > 0.3) the cortical glucose oxidation in control mice. The improvement in energy metabolism with RS intervention was relatively higher in the subcortical regions (∼40–70%; striatum and hippocampus) as compared with that in the cerebral cortex (∼20–30%) of AlCl_3_ treated mice. These data indicate beneficial effect of RS on the energy metabolism of neurons in AlCl_3_ treated mice.

## Discussion

Although brain energy metabolism has been studied extensively in qualitative manner but its quantitative significance is not much explored in AD. In the present study, we evaluated the excitatory and inhibitory neuronal activity in several brain regions like cerebral cortex, hippocampus, and striatum in the AlCl_3_ model of AD. AlCl_3_ at doses ranging from 1 to 500 mg/kg has been used in different studies in animals to mimic AD physiology (Walton, 2007; Chen et al., 2013). The dose of AlCl_3_ (40 mg/kg) used in the current study is in the intermediate range of that used in a previous study (Shati et al., 2011). In addition, effects of RS, an Ayurvedic formulation, on the brain energy metabolism in AlCl_3_ model of AD was examined.

Chronic administration of aluminum chloride in rodent has been used to model the AD in animals. Exposure of AlCl_3_ (50 mg/kg) to albino mice for 3 months period showed significant impairment of spatial working memory as compared with controls (Rebai and Djebli, 2008). In a more recent study, in addition to reduced working memory, a significant accumulation of Aβ plaques in the cerebral cortex and hippocampus of mice exposed to AlCl_3_ was also shown (Chen et al., 2013). In the present study, we have administered AlCl_3_ (40 mg/kg) intraperitoneally for 60 days, and memory and cerebral metabolism were assessed on 67^th^ day or latter (**Figure 2A** Group **I**). These animals showed significant reduction in the memory and neurometabolic activity. Moreover, immuno-histochemical analysis exhibited presence of small Aβ plaques in the cerebral cortex and hippocampus of the AlCl_3_ treated mice. In the second set of experiments, AlCl_3_ (40 mg/kg) was administered orally to mice for 60 days, and behavioral and metabolic analysis were undertaken beyond 97^th^ day, i.e., ∼35 days after the last treatment of AlCl_3_ (**Figure 2A** Group **II**). As shown in **Figures 7B** and **8**, these mice showed impairment in memory and metabolic activity. It should be noted that a waiting period of 35 days further deteriorated neurometabolic activity in AlCl_3_ mice. These data suggest that impact of AlCl_3_ on memory and cerebral metabolic activity lasted at least ∼5–6 weeks of AlCl_3_ withdrawal. However, the long-term effect of AlCl_3_ withdrawal on memory and metabolic function need further investigation.

Measurements of levels of brain metabolites in AD patients using ^1^H Magnetic Resonance Spectroscopy have indicated a decrease in levels of NAA (Kantarci et al., 2003) and glutamate (Hattori et al., 2002), and an increase in myo-inositol level (Antuono et al., 2001; Wang J. et al., 2010; Gordon et al., 2012). The decrease in NAA level has been attributed with neuronal death and reduced viability of neurons, while the increased level of myo-inositol is associated with astrogliosis. Significant increase in the level of myo-inositol and reduction in the levels of glutamate and NAA, a neuronal marker, have been reported in AβPP-PS1 mice beyond 12 months of age (Marjanska et al., 2005). AβPP-PS1 mice display sparse plaque loading and unperturbed neurometabolites homeostasis at the age of 6 months that represents preclinical stage of AD (Holcomb et al., 1998). We did not find any significant change in the level of neurometabolites the cerebral cortex and hippocampus of AlCl_3_ treated mice. However, a significant reduction in the level of glutamate and NAA was observed in the striatum of AlCl_3_ treated mice. Our findings of unperturbed neurometabolites homeostasis in the cerebral cortex and hippocampus suggest that AlCl_3_ treated mice might represent preclinical stage of AD. Furthermore, the finding of increased level of myo-inositol is suggestive of enhanced astroglial activity in the hippocampus of AlCl_3_ exposed mice.

Cerebral glucose metabolism has been evaluated using positron emission tomography in AD patients. Hypo-glucose metabolism has been reported in patients at the various stages of AD or even before the appearance of clinical symptoms (Drzezga et al., 2003; Kim et al., 2005). In consistence with these observations, decreased TCA cycle flux for glutamatergic and GABAergic neurons was reported in the frontal cortex and hippocampal formation of transgenic McGill-R-Thy1-APP rat model of AD (Nilsen et al., 2014). The reduction in brain energy metabolism was also observed at the age of 13 months in a triple transgenic mouse harboring APP(Swe), PS1(M146V), and Tau(P301L) transgenes (Sancheti et al., 2014). Moreover, impaired glutamatergic and GABAergic metabolic activity has been observed in the cerebral cortex, hippocampus, and striatum even at the age of 6 months in AβPP-PS1 mice (Tiwari and Patel, 2012). The data from the current study indicate that AlCl_3_ treatment in mice exhibit reduction in energy metabolism of glutamatergic (15–27%) and GABAergic neurons (8–27%) across brain (**Figure 6**). Furthermore, analysis carried out one month after AlCl_3_ treatment indicated severe metabolic deficit in glutamatergic (18–34%) and GABAergic (24–49%) neurons across brain (**Figure 8**). These data suggest the AlCl_3_ affects the neuronal metabolic activity in time-dependent manner. As there was no significant perturbation in the level of glutamate, GABA, and NAA in the cerebral cortex and hippocampus, these mice resemble to the preclinical stage of AD.

Neurons and astrocytes work in a concerted manner to bring about optimal neurotransmitter cycling and functioning of the brain. Perturbation in the flux through glutamate and GABA neurotransmitter pathways is associated with several psychiatric and neurological conditions. ^13^C NMR spectroscopy together with infusion of [1,6-^13^C_2_]glucose provides a unique approach for monitoring neuronal activity under different pathological conditions (Patel et al., 2004) and during interventions (Bagga et al., 2013; Sancheti et al., 2014). The trafficking of neurotransmitters amino acids between neurons and astrocytes, commonly referred as neurotransmitter cycling, plays important role in the normal functioning of brain. As per our knowledge, there is no quantitative information for the neurotransmitter flux in AD patients. Qualitative analysis of ^13^C label incorporation into [4-^13^C]glutamine from [1-^13^C]glucose in the transgenic mouse model suggested perturbation in glutamate–glutamine cycling in AD condition (Nilsen et al., 2014). A very recent study conducted using double transgenic AβPP-PS1 mice has indicated severe reduction in excitatory and inhibitory neuronal metabolic activity and neurotransmitter cycling fluxes, and increased astroglial metabolic activity across brain (Patel et al., 2017). The quantitative analysis of neurotransmitter cycling flux in the current study indicated that exposure of AlCl_3_ decreased the trafficking of neurotransmitter between neurons and astrocytes (**Figure 6B**) in the cerebral cortex (27%), hippocampus (16%), and striatum (14%). The decrease in neurotransmitter cycling flux in the cerebral cortex and hippocampus was contributed by deficit in both glutamate–glutamine and GABA–glutamine cycle, while it was mostly due to glutamate–glutamine cycling in striatum (**Figures 6D,F**). Hence, the preclinical stage of AD is manifested by reduced excitatory and inhibitory activity in the cerebral cortex and hippocampus, and selective reduction in excitatory function in the striatum. The cerebral cortex plays an important role in higher order cognition, while the hippocampus is involved in the learning and memory. The reduced neuronal activity in the cerebral cortex and hippocampus in AlCl_3_ exposed mice point toward impairment of cognitive function and memory at preclinical stage of AD.

The treatment strategies for AD have focused on decreasing the load of β-amyloid by inhibition of β-/γ-secretases and Aβ oligomerization; activation of proteases, α-secretase, and immunotherapy (Auld et al., 2002; Citron, 2010; Götz et al., 2012). Although different approaches have been used to combat AD, there is very limited success for the treatment of the disease. Donepezil, an acetylcholine esterase inhibitor, is the first line FDA approved drug for the palliative treatment of AD. It is used to improve cognitive function in AD patients but does not slow the progression of the disease. Hence, there is an increased effort for screening of traditional formulations for the treatments of AD.

In recent time, interest in using plant products and other traditional therapies for alleviation of symptoms of AD in animal models and human patients has increased. Preparation based on different plants such as *Amalaki Rasayana* (Tiwari et al., 2017), *Withania somnifera* (Sehgal et al., 2012), *Centellaasiatica* (Dhanasekaran et al., 2009), *Huperzine A* (Wang and Tang, 2005), *Gingko biloba* (Oken et al., 1998), *Curcumin* (Wang H.M. et al., 2010), and *Melissa officinalis* (Iwasaki et al., 2005) have been shown to reduce dementia with lesser side effects than conventional drugs, and therefore, regarded as potential anti-AD drugs (Sun et al., 2013). Extract of the roots of *Withania somnifera* was shown to reverse the accumulation of β-amyloid peptides (Aβ) and oligomers, and plaque pathology in the young and middle age of AβPP-PS1 transgenic mouse model of AD (Sehgal et al., 2012). The protective effect of RS has been examined in *GMR-Aβ42 Drosophila* model of AD. The transgenic *Drosophila* larvae reared on RS supplemented food exhibited substantial reduction in the damage to eye disk, and amyloid Aβ plaques compared to those reared on normal food (Dwivedi et al., 2013). This suggests that RS has the potential to ameliorate AD condition by reducing Aβ plaques. Although Aβ plaque load has not been examined in the RS treated AlCl_3_ mice in the current study, it is presumed that a similar mechanism may be operating in mice also, which may be examined in further studies. Our data suggest that intervention with RS completely restores the memory of mice exposed to AlCl_3_. Most interestingly, RS intervention resulted in improved functionality of neuronal cells as revealed by the cerebral metabolic rate of neuronal glucose oxidation. The metabolic activity of glutamatergic and GABAergic neurons that was impaired drastically in AlCl_3_ treated mice was restored very close to control levels across the brain. The neuronal glucose oxidation and neurotransmitter cycling flux have been shown to be stoichiometrically coupled (Sibson et al., 1998; Patel et al., 2004). Hence, the finding of increased neuronal glucose oxidation with RS intervention in AlCl_3_ treated mice is suggestive of increased glutamate–glutamine and GABA–glutamine neurotransmitter cycling flux with RS treatments.

A common objection against metal-derived Ayurvedic formulations is their potential toxicity (Nishteswar, 2013). In this context, it is significant to note that as reported earlier (Dwivedi et al., 2012; Ramanan et al., 2015), we did not find any evidence for toxicity resulting from RS administration to control mice (**Figure 8**). Moreover, analysis of mercury indicated no significant (F[2,9] = 0.27, *p* = 0.77) change in mercury level in RS treated mice (Supplementary Figure 1). The control group of mice did not suffer any negative consequences, while the experimental group showed positive improvement in memory functions and brain energy metabolism.

## Limitations

There are a few potential limitations of the study. The first among them is the use of ratio of neurotransmitter cycling to the tri-carboxylic acid cycle (V_cyc_/V_TCA_) for glutamatergic and GABAergic neurons for the constraint during model fitting to previously reported value (Tiwari et al., 2013). In the current study, we have used the ratio (V_cyc_/V_TCA_) that was obtained in normal mice (Tiwari et al., 2013). These values have potential to be altered following AlCl_3_ exposure in mice, and hence the flux of neurotransmitter cycling and TCA cycle of glutamatergic and GABAergic neurons. Previous analysis has indicated that a 50% reduction in the value of ratio of neurotransmitter cycling to TCA cycle rate decreased the TCA cycle and neurotransmitter cycling at most by 7%, while an increase in the ratio results in 14% higher metabolic rates (Wang J. et al., 2010). Therefore, we believe that even if AlCl_3_ neurotoxicity changes the V_cyc_/V_tca_ ratio, it will not have much impact on the conclusion of the study. The ratio of neurotransmitter cycle to neuronal TCA cycle flux could be pinned down more accurately by using a steady state infusion of [2-^13^C]acetate, respectively (Patel et al., 2005; Tiwari et al., 2013).

Metabolic rates in the second set of study are based on the trapping of ^13^C label in different neurometabolites such as glutamate, GABA and aspartate, using Equations 1–3. This approach does not account for the flow of label into glutamine-C4 via neurotransmitter cycling. Moreover, ^13^C label lost as CO_2_, and shuttled into lactate-C3 and alanine-C3 were not accounted. Hence, the cerebral metabolic rates of glucose oxidation derived using Equations 1–3 will be underestimated. However, it may be argued that underestimates in metabolic rates would be of similar degrees in the different groups. Hence, the relative values, and direction of the changes in their respective CMR_Glc_ would remain the same, and thus not have much impact on the findings. More accurate estimates of cerebral metabolic rates of glucose oxidation by GABAergic and glutamatergic neuronal TCA and neurotransmitter cycles could be obtained by modeling of ^13^C turnover of amino acids from [1,6-^13^C_2_]glucose as described for the first set of experiment.

## Conclusion

Exposure of AlCl_3_ results in perturbation in neurometabolism across brain without severely affecting the homeostasis of neurometabolites in the cerebral cortex and hippocampus, an observation similar to preclinical stage in AβPP-PS1 mice (Tiwari and Patel, 2012). Hence, AlCl_3_ exposure paradigm may be useful to model preclinical stage of AD. Our finding that intervention with RS improved memory and neuronal metabolism in AlCl_3_ treated mice indicates that RS may be useful for the management of memory and cognitive functions in preclinical stage of AD.

## Author Contributions

KS performed study, analyzed data, prepared figures, wrote the paper; NR: performed study; PV: performed study; VT: performed study; RR: performed study; SL: interpreted data, wrote the paper; AP: designed research, analyzed data, interpreted data, prepared figures, wrote the paper, supervised and directed the overall project. All authors reviewed the manuscript.

## Conflict of Interest Statement

The authors declare that the research was conducted in the absence of any commercial or financial relationships that could be construed as a potential conflict of interest.
