# Supplementary material for: Energetics of Excitatory and Inhibitory Neurotransmission in Aluminum Chloride Model of Alzheimer’s Disease: Reversal of Behavioral and Metabolic Deficits by Rasa Sindoor
Source: Front Mol Neurosci. 2017 Oct 17;10:323. doi: 10.3389/fnmol.2017.00323 (PMC5651029; doi:10.3389/fnmol.2017.00323)

Supplementary Materials

Energetics of Excitatory and Inhibitory Neurotransmission in Aluminum Chloride Model of Alzheimer's Disease: Reversal of Behavioral and Metabolic Deficits by Rasa Sindoor

Kamal Saba[1], Niharika Rajnala[1], Pandichelvam Veeraiah[1], Vivek Tiwari[1], Rohit Kumar Rana[2], Subhash C. Lakhotia[3], Anant B. Patel[1*]

[1]*NMR Microimaging and Spectroscopy, CSIR-Centre for Cellular and Molecular Biology, Uppal Road, Hyderabad, India;* [2]*CSIR-Indian Institute of Chemical Technolog*y, Tarnaka, Hyderabad, India; [3]*Cytogenetics Lab, Department of Zoology, Banaras Hindu University, Varanasi, India*

***Correspondence Address:**
Dr. Anant B. Patel, NMR Microimaging and Spectroscopy, Centre for Cellular and Molecular Biology, Uppal Road, Habsiguda, Hyderabad 500007, India
Telephone: +91-40-27192838
Fax: +91-40-27160591
Email: abpatel@ccmb.res.in

Supplementary Table 1 Mass and isotope balance equations with various rates and pool size for the cerebral cortex

Mass Balance Equations:

$dGlc_{brain}/dt = V_{max(in)}\ Glc_{blood}/(K_{m(in)} + Glc_{blood}) – V_{max(out)}\ Glc_{brain}\ /(K_{M(out)} + Glc_{brain}) + CMR_{glc}$

$dLac/dt = dGlu_{Glu}/dt = dGlu_{GABA}/dt = dGlu_{A}/dt = dGABA/dt = dGln/dt = dAsp_{Glu}/dt = dAsp_{GABA}/dt = dAsp_{A}/dt = dLac/dt = dKG_{Glu}/dt = dKG_{GABA}/dt = dKG_{A}/dt = dOAA_{Glu}/dt = dOAA_{GABA}/dt = dOAA_{A}/dt = 0$

Isotope Balance Equations:

$d(Glc_{brain,C16})/dt = V_{max(in)}\ (Glc_{blood,C16})/\ (K_{m(in)} + Glc_{blood}) - V_{max(out)}\ (Glc_{brain,C16})/(Km_{out} + Glc_{brain}) - CMR_{glc}\ (Glc_{brain,C16}/Glc_{brain})$

$dLac_{C3}/dt = 2\ CMR_{glc}(Glc_{brain,C16}/Glc_{brain}) + V_{dil(Lac)}\ (0) - (V_{pc} + V_{dil(Lac)} + V_{pdh(A)} + V_{pdh(GABA)} + V_{pdh(Glu)})\ (Lac_{C3}/Lac)$

$dGlu_{Glu,C4}/dt = V_{cyc(Glu-Gln)}\ (Gln_{C4}/Gln) + V_{xGlu(KG-Glu)}\ (KG_{Glu,C4}/KG_{Glu}) - (V_{cyc(Glu-Gln)} + V_{xGlu(Glu-KG)})\ (Glu_{Glu,C4}/Glu_{(Glu)})$

$dGlu_{A,C4}/dt = V_{cyc(Glu-Gln)}\ (Glu_{Glu,C4}/Glu_{(Glu)}) + V_{xA(KG-Glu)}\ (KG_{A,C4)}/KG_{A}) + V_{cyc(GABA-Gln)}\ (KG_{A,C4}/KG_{A}) - (V_{gln} + V_{xA(Glu-KG)})\ (Glu_{A,C4}/Glu_{A})$

$dGln_{4}/dt = V_{gln}\ (Glu_{A,C4}/Glu_{A}) + V_{dilGln}\ (0) - [V_{efflux} + V_{cyc(Glu-Gln)} + V_{cyc(GABA-Gln)}](Gln_{4}/Gln)$

$dKG_{A,C4}/dt = V_{pdh(A)}\ (Lac_{C3}/Lac) + V_{xA(Glu-KG)}\ (Glu_{A,C4}/Glu_{A}) + V_{dilA}\ (0) - (V_{tca(ANet)} + V_{xA(KGGlu)} + V_{cyc(GABA-Gln})\ (KG_{A,C4}\ /KG_{A})$

$dKG_{A,C3}/dt = V_{xA(Glu-KG)}\ (Glu_{A,C3}/Glu_{A}) + V_{tca(A)}\ (OAA_{A,C2}/OAA_{A}) - (V_{tca(ANet)} + V_{xA(KG-Glu)} + V_{cyc(GABA-Gln)})\ (KG_{A,C3}/KG_{A})$

$dGlu_{A,C3}/dt = V_{xA(KG-Glu)}\ (KG_{A,C3}/KG_{A}) + V_{cyc(Glu-Gln)}\ (Glu_{Glu,C3}/Glu_{Glu}) + V_{cyc(GABA-Gln)}\ (K_{GA,C3}/K_{GA}) - (V_{gln} + V_{xA(Glu-KG})\ (Glu_{A,C3}/Glu_{A})$

$dGln_{C3}/dt = V_{gln}\ (Glu_{A,C3}/Glu_{A}) + V_{dilGln}\ (0) - [V_{efflux} + V_{cyc(Glu-Gln)} + V_{cyc(GABA-Gln)}](Gln_{C3}/Gln)$

$dGlu_{Glu,C3}/dt = V_{cyc(Glu-Gln)}\ (Gln_{C3}/Gln) + V_{xGlu(KG-Glu)}\ (KG_{Glu,C3}/KG_{Glu}) - (V_{cyc(Glu-Gln)} + V_{xGlu(Glu-KG)})\ (Glu_{Glu,C3}/Glu_{Glu})$

$dKG_{Glu,C4}/dt = V_{xGlu(Glu-KG)}\ (Glu_{Glu,C4}/Glu_{Glu}) + V_{dil(Glu)}\ (0) + V_{pdh(Glu)}\ (Lac_{C3}/Lac) - (V_{xGlu(KG-Glu)} + V_{tca(Glu)})\ (KG_{Glu,C4}/KG_{Glu})$

$dKG_{Glu,C3}/dt = V_{xGlu(Glu-KG)}\ (Glu_{Glu,C3}/Glu_{Glu}) + V_{tca(Glu)}\ (OAA_{Glu,C2}/OAA_{Glu}) - (V_{xGlu(KG-Glu)} + V_{tca(Glu)})\ (KG_{Glu,C3}/KG_{Glu})$

$dAsp_{Glu,C3}/dt = V_{xGlu(OAA-Asp)} (OAA_{Glu,C2}/OAA_{Glu}) - V_{xGlu(Asp-OAA)} (Asp_{Glu,C3}/Asp_{Glu})$

$dAsp_{A,C3}/dt = V_{xA(OAA-Asp)} (OAA_{A,C2}/OAA_{A}) - V_{xA(Asp-OAA)} (Asp_{A,C3}/Asp_{A})$

$dOAA_{Glu,C2}/dt = V_{xGlu(Asp-OAA)} (Asp_{Glu,C3}/Asp_{Glu}) + 0.5 V_{tca(Glu)} (KG_{Glu,C4}/KG_{Glu}) + 0.5 V_{tca(Glu)} (KG_{Glu,C3}/KG_{Glu}) - [V_{xGlu(OAA-Asp)} + V_{tca(Glu)}] (OAA_{Glu,C2}/OAAGlu)$

$dOAA_{A,C2}/dt = 0.5 V_{tcaANet} (KG_{A,C4}/KG_{A}) + 0.5 V_{tcaANet} (KG_{A,C3}/KG_{A}) + V_{xA(Asp-OAA)} (Asp_{A,C3}/Asp_{A}) + 0.5 V_{pc} (Lac_{C3}/Lac) + V_{cyc(GABA-Gln)} (GABA_{C2}/GABA) - [V_{xA(OAA-Asp)} + V_{tcaA}] (OAA_{A,C2}/OAA_{A})$

$dGABA_{C2}/dt = V_{gad} (Glu_{GABA,C4}/Glu_{GABA}) - (V_{shunt} + V_{cyc(GABA-Gln)}) (GABA_{C2}/GABA)$

$dKG_{GABA,C4}/dt = V_{pdhGABA} (Lac_{C3}/Lac) + V_{xGABA(Glu-KG)} (Glu_{GABA,C4}/Glu_{GABA}) + V_{dilGABA} (0) - [V_{tcaGABANet} + V_{xGABA(KG-Glu}] (KG_{GA,C4}/KG_{GABA})$

$dGlu_{GABA,C4}/dt = V_{cyc(GABA-Gln)} (Gln_{C4}/Gln) + V_{xGABA(KG-Glu)} (KG_{GABA,C4}/KG_{GABA}) - (V_{gad} + V_{xGABA(Glu-KG)}) (Glu_{GABA,C4}/Glu_{GABA})$

$dKG_{GABA,C3}/dt = V_{xGABA(Glu-KG)} (Glu_{GABA,C3}/Glu_{GABA}) + V_{tca(GABA)} (OAA_{GABA,C3}/OAA_{GABA}) - (V_{tca(GABANet)} + V_{xGABA(KG-Glu)}) (KG_{GABA,C3}/KG_{GABA})$

$dGABA_{C3}/dt = V_{gad} (Glu_{GABA,C3}/Glu_{GABA}) - (V_{shunt} + V_{cyc(GABA-Gln)}) (GABA_{C3}/GABA)$

$dGlu_{GABA,C3}/dt = V_{cyc(GABA-Gln)} (Gln_{C3}/Gln) + V_{xGABA(KG-GABA)} (KG_{GABA,C3}/KG_{GABA}) - [V_{gad} + V_{xGABA(Glu-KG)}] (Glu_{GABA,C3}/Glu_{GABA})$

$dAsp_{GABA,C3}/dt = V_{xGABA(OAA-Asp)} (OAA_{GABA,C2}/OAA_{GABA}) - V_{xGABA(Asp-OAA)} (Asp_{GABA,C3}/Asp_{GABA})$

$dOAA_{Glu,C3}/dt = V_{xGlu(Asp-OAA)} (Asp_{Glu,C3}/Asp_{Glu}) + 0.5 V_{tca(Glu)} (KG_{Glu,C3}/KG_{Glu}) + 0.5V_{tca(Glu)} (KG_{Glu,C4}/KG_{Glu}) - [V_{xGlu(OAA-Asp)} + V_{tca(Glu)}] (OAA_{Glu,C3}/OAA_{Glu})$

$dOAA_{A,C3}/dt = V_{cyc(GABA-Gln)} (GABA_{A,C3}/GABA) + V_{xA(Asp-OAA)} (Asp_{A,C3}/Asp_{A}) + 0.5 V_{pc} (Lac_{C3}/Lac) + 0.5 V_{tcaANet} (KG_{A,C4}/KG_{A}) + 0.5 V_{tcaANet} (KG_{A,C3}/KG_{A}) - [V_{xA(OAA-Asp)} + V_{tcaA}] (OAA_{A,C3}/OAA_{A})$

$dAsp_{A,C2}/dt = V_{xA(OAA-Asp)} (OAA_{A,C3}/OAA_{A}) – V_{xA(Asp-OAA)} (Asp_{A,C2}/Asp_{A})$

$dAsp_{Glu,C2}/dt = V_{xGlu(OAA-Asp)} (OAA_{Glu,C3}/OAA_{Glu}) – V_{xGlu(Asp-OAA)} (Asp_{Glu,C2}/Asp_{Glu})$

$dAsp_{GABA,C2}/dt = V_{xGABA(OAA-Asp)} (OAA_{GABA,C3}/OAA_{GABA}) – V_{xGABA(Asp-OAA)} (Asp_{GABA,C2}/Asp_{GABA})$

$dOAA_{GABA,C2} /dt = 0.5 V_{tca(GABANet)} ((KG_{GABA,C3}/KG_{GABA}) + (KG_{GABA,C4}/KG_{GABA})) + 0.5 V_{shunt} \{(GABA_{C3}/GABA) + (GABA_{C2}/GABA)\} + V_{xGABA(Asp-OAA)} (Asp_{GABA,C3}/Asp_{GABA}) - (V_{tca(GABA)} + V_{xGABA(OAA-Asp)}) (OAA_{GABA,C2}/OAA_{GABA})$

$dOAA_{GABA,C3}/dt = 0.5\ V_{tca(GABANet)}\ ((KG_{GABA,C3}\ /KG_{GABA}) + (KG_{GABA,C4}/KG_{GABA})) + 0.5\ V_{shunt}\ \{(GABA_{C2}/GABA) + (GABA_{C3}/GABA)\} + V_{xGABA(Asp\text{-}OAA)}\ (Asp_{GABA,C2}/Asp_{GABA}) - (V_{tca(GABA)} + V_{xGABA(OAA\text{-}Asp)})\ (OAA_{GABA,C3}\ /OAA_{GABA})$

Distribution of Glutamate and Aspartate in Different Compartments [4]:

		Glutamatergic Neurons	GABAergic Neurons	Astrocyte
Glutamate	Cerebral Cortex	0.82	0.02	0.16
	Hippocampus	0.86	0.02	0.12
	Striatum	0.83	0.02	0.15
Aspartate	Cerebral Cortex	0.42	0.42	0.16
	Hippocampus	0.44	0.44	0.12
	Striatum	0.425	0.425	0.15

Pool Concentrations:

Glc_{Brain} (Concentration of brain glucose) = $Km_{(out)}$ X2 / ($V_{max(out)}$ - X2) = 3.72 µmol/g

Glu_{Total} = 12.29 µmol/g (measured)

Glu_A (Concentration of Glu in Astrocytes) =1.9664 µmol/g

Glu_{GABA} (Concentration of Glu in GABAergic neurons) = 0.2458 µmol/g [1,2]

Glu_{Glu} (Concentration of Glu in Glutamatergic neurons) = 10.0778 µmol/g

GABA = 2.57 µmol/g (measured)

Gln = 5.84 µmol/giv(measured)

Asp_{Total} = 2.11 µmol/g (measured)

Asp_A (Concentration of Asp in astrocytes) = 0.3376 µmol/g

Asp_{GABA} (Concentration of Asp in GABAergic neurons)= 0.8862 µmol/g

Asp_{Glu} (Concentration of Asp in Glutamatergic neurons) = 0.8862 µmol/g

KG_A (Concentration of α-KG in astrocytes) = 0.09 µmol/g

KG_{Glu} (Concentration of α-KG in Glutamatergic neurons) = 0.1 µmol/g

KG_{GABA} (Concentration of α-KG in GABAergic neurons) = 0.01 µmol/g [3]

Lac = 1.5 μmol/g

OAA_{Total} = 0.2 μmol/g

OAA_A (Concentration of OAA in astrocytes)= OAATotal-OAAN-OAAGA = 0.05 μmol/g

OAA_{GABA} (Concentration of OAA in GABAergic neurons) = 0.05 μmol/g [3]

OAA_{Glu} (Concentration of OAA in Glutamatergic neurons)= 0.1 μmol/g [3]

Values of Rates:

$K_{m(in)}$ (Michaelis−Menten half-saturation constant for blood-to-brain glucose transport) = 13.9 mM [4]

$K_{m(out)}$ (Michaelis−Menten half-saturation constant for brain-to-blood glucose transport) = $K_{m(in)}$ x Vd = 10.7 μmol/g [4]

$V_{max(in)}$ (Michaelis–Menten maximum uptake rate for blood-to-brain glucose transport) = 5.8 x CMR_{glc} = 2.44 μmol/min/g [4]

$V_{max(out)}$ (Michaelis–Menten maximum uptake rate for brain-to-blood glucose transport) = $V_{max(in)}$ = 2.44 μmol/min/g

$V_{cyc(Glu\text{-}Gln)}/V_{tca(Glu)}$ = 0.39 [5]

$V_{cyc(GABA\text{-}Gln)}/V_{tca(GABA)}$ = 0.43 [5]

V_d (space for brain water) = 0.77 ml/g [6]

V_{dilGln} (Rate of dilution of glutamine from blood) = 0.001 μmol/min/g ***(iterated)***

V_{efflux} (Rate of glutamine efflux from brain) = V_{pc} + V_{dilGln} = 0.07 μmol/min/g

V_{pc} (Rate of pyruvate carboxylation) = 0.069 μmol/min/g ***(iterated)***

$V_{tca(ANet)}$) = 0.12 μmol/min/g ***(iterated)***

$V_{tca(A)}$ (Astrocytic TCA cycle flux) = $V_{tca(ANet)}$ + V_{gln} - $V_{cyc(Glu\text{-}Gln)}$ = 0.055 μmol/min/g

V_{dilA} (Rate of dilution of astrocytic pyruvate/lactate from blood) = 0.155 μmol/min/g ***(iterated)***

$V_{pdh(A)}$ (Rate of pyruvate dehydorgenase in astrocyctes) = $V_{tca(A)}$ - V_{dilA} = 0.067 μmol/min/g

$V_{tca(GABANet)}$ = 0.225 μmol/min/g ***(iterated)***

V_{shunt} (Flux of GABA shunt) = 0.003 μmol/min/g ***(iterated)***

$V_{tca(GABA)}$ (TCA cycle flux of GABAergic neurons) = $V_{tca(GABANet)}$ + V_{shunt} = 0.228 μmol/min/g

$V_{cyc(GABA-Gln)}$ (GABA-Gln cycle flux) = {$V_{cyc(GABA-Gln)}/V_{tca(GABA)}$} x $V_{tca(GABA)}$ = 0.098 µmol/min/g ***(calculated)***

$V_{dil(GABA)}$ (Rate of dilution of GABAergic pyruvate/lactate from blood) = 0.014 µmol/min/g ***(iterated)***

$V_{pdh(GABA)}$ (Rate of pyruvate dehydorgenase in GABAergic neurons) = $V_{tca(GABA)}$ - $V_{dil(GABA)}$ = 0.214 µmol/min/g

V_{gad} (Rate of GABA synthesis) = V_{shunt} + $V_{cyc(GABA-Gln)}$ = 0.102 µmol/min/g

$V_{tca(Glu)}$ (TCA cycle flux of Glutamatergic neurons) = 0.533 µmol/min/g ***(iterated)***

$V_{dil(Glu)}$ (Rate of dilution of pyruvate/lactate in glutamatergic neurons from blood)= 0.040 µmol/min/g ***(iterated)***

$V_{pdh(Glu)}$ (Rate of pyruvate dehydorgenase in Glutamatergic neurons) = $V_{tca(Glu)}$ - $V_{dil(Glu)}$ = 0.493 µmol/min/g

$CMR_{glc} = (V_{pdh(Glu)} + V_{pdh(GABA)} + V_{pdh(A)} + V_{pc}) / 2$

$V_{cyc(Glu-Gln)}$ (Glu-Gln cycle flux) = {$V_{cyc(Glu-Gln)}/V_{tca(Glu)}$} x $V_{tca(Glu)}$ = 0.208 µmol/min/g ***(calculated)***

V_{gln} (rate of glutamine synthesis) = $V_{cyc(Glu-Gln)}$ + $V_{cyc(GABA-Gln)}$ + V_{pc}

$V_{xA(Glu-KG)}$ (Glu and α-KG exchange flux in astrocytes) = 1.00 µmol/min/g

$V_{xA(Asp-OAA)}$ (Asp and OAA exchange flux in astrocytes) = $V_{xA(OAA-Asp)}$ = $V_{xA(Glu-KG)}$ = 1.00 µmol/min/g

$V_{xA(KG-Glu)}$ (α-KG and Glu exchange flux in astrocytes) = $V_{xA(Glu-KG)}$ + V_{pc} = 1.068 µmol/min/g

$V_{xGABA(Glu-KG)}$ (Glu and α-KG exchange flux in GABAergic neurons) = 0.503 µmol/min/g

$V_{xGABA(Asp-OAA)}$ (Asp and OAA exchange flux in GABAergic neurons) = $V_{xGABA(OAA-Asp)}$ = $V_{xGABA(Glu-KG)}$ = 0.50 µmol/min/g

$V_{xGABA(KG-Glu)}$ (α-KG and Glu exchange flux in GABAergic neurons) = $V_{xGABA(Glu-KG)}$ + V_{gad} - $V_{cyc(GABA-Gln)}$ = 0.503 µmol/min/g

$V_{xGlu(Glu-KG)}$ (Glu and α-KG exchange flux in glutamatergic neurons) = 1000 µmol/min/g ***(iterated)***

$V_{xGlu(OAA-Asp)}$ (OAA and Asp exchange flux in glutamatergic neurons) = $V_{xGlu(Asp-OAA)}$ = $V_{xGlu(KG-Glu)}$ = $V_{xGlu(Glu-KG)}$ = 1000 µmol/min/g

$X_1 = V_{max(in)}\ Blood_{(Glucose)}/(K_{m(in)}+Blood_{(Glucose)})$ = 1.051 µmol/min/g

$X_2 = X_1-CMR_{glc}$ = 0.630 µmol/min/g

V_{cyc}/V_{TCA} for Glutamatergic and GABAergic neuron in different brain region [4]:

	Glutamatergic Neurons	GABAergic Neurons
Cerebral Cortex	0.39±0.04	0.43±0.02
Hippocampus	0.49±0.03	0.35±0.02
Striatum	0.24±0.03	0.43±0.02

References

1. Patel AJ, Johnson AL, Balazs R: Metabolic compartmentation of glutamate associated with the formation of gamma-aminobutyrate. *J Neurochem* 1974, **23**:1271-1279

2. Storm-Mathisen J, Leknes AK, Bore AT, Vaaland JL, Edminson P, Haug, FM, Ottersen OP: First visualization of glutamate and GABA in neurons by immunocytochemistry. *Nature* 1983, **301**:517-520

3. Hawkins RA, Mans AM: Intermediary metabolism of carboyhdrates and other fuels. I. In: *Biochem J* (Lajtha A ed) Plenum Press, New York 1983, **122**:259-294

4. Mason GF, Rothman DL, Behar KL, Shulman RG: NMR determination of the TCA cycle rate and alpha-ketoglutarate/glutamate exchange rate in rat brain. *J Cereb Blood Flow Metab* 1992, **12**:434–447.

5. Tiwari V, Ambadipudi S, Patel AB: Glutamatergic and GABAergic TCA cycle and neurotransmitter cycling fluxes in different regions of mouse brain. *J Cereb Blood Flow Metab* 2014 **33**:1523-1531.

6. Gjedde A, Diemer NH: Autoradiographic determination of regional brain glucose content. *J Cereb Blood Flow Metab 1983,* **3**:303–310

Supplementary Table 2 Concentration (µmol/g) of neuro-metabolites in $AlCl_3$-treated mice

Brain Region	Treatment Group	Neurometabolites							
		Glu	GABA	Gln	Asp	NAA	m-Ino	Tau	Cho
Cerebral Cortex	NS (n=12)	12.1±0.3	2.6±0.1	5.8±0.4	2.1±0.1	7.4±0.8	6.4±0.1	11.9±0.3	1.8± 0.1
	$AlCl_3$ (n=12)	12.3±0.3	2.7±0.1	6.2±0.3	2.2±0.2	8.0±0.2	6.7±0.3	12.2±0.4	1.9±0.1
Hippocampus	NS (n=12)	9.9±0.3	3.6±0.1	4.6±0.2	1.6±0.1	6.4±0.6	6.3± 0.2	9.7±0.2	1.7±0.1
	$AlCl_3$ (n=12)	10.5±0.2	3.8±0.3	5.0±0.2	1.9±0.1	7.2±0.2	7.5±0.3**	10.3±0.4	2.0±0.1
Striatum	NS (n=12)	10.9±0.4	5.9±0.3	5.9±0.4	1.7±0.1	7.9±0.3	7.5± 0.2	11.4±0.5	2.4±0.1
	$AlCl_3$ (n=12)	9.6±0.2*	6.3±0.3	6.0±0.3	1.8±0.1	7.1±0.3*	7.3±0.4	10.2±0.6	2.2±0.2

Concentration of metabolites was measured in non-edited ^{1}H-[^{13}C]-NMR spectrum of tissue extracts using [2-^{13}C]glycine as standard. Values are represented as mean±SEM. * $p<0.01$, ** $p<0.001$ when $AlCl_3$ treated mice were compared with normal saline treated controls. NS, normal saline.

Supplementary Figure 1 The level of mercury in $AlCl_3$ and RS treated mice. The mecrury level was measured in cortical homogenate using inductively coupled plasma mass spectrometry (ICP-MS.

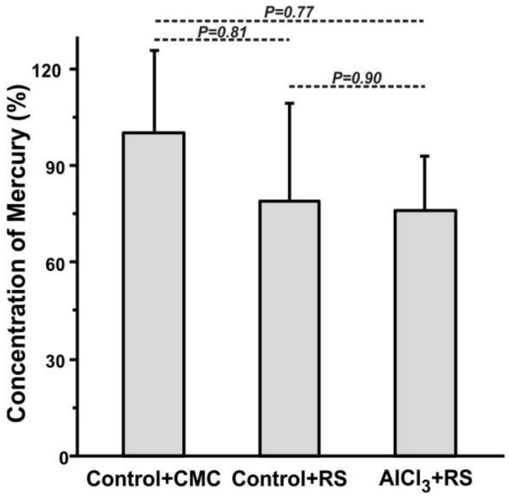

Supplementary Figure 2 The ^{1}H-[^{13}C]-NMR spectra from mouse cerebral cortex showing ^{13}C labeling of amino acids from [1,6-$^{13}C_2$]glucose. Mice were administered Rasa Sindoor (2 g/kg, intragrastric) for 30 days in $AlCl_3$ treated mice (40 mg/kg, intragastric). Mice were infused with [1,6-$^{13}C_2$]glucose for 10 min, and cortical metabolites were extracted using ethanol extraction protocol. The ^{1}H-[^{13}C]-NMR spectra of cortical extracts were recorded using 600 MHz NMR spectrometer.

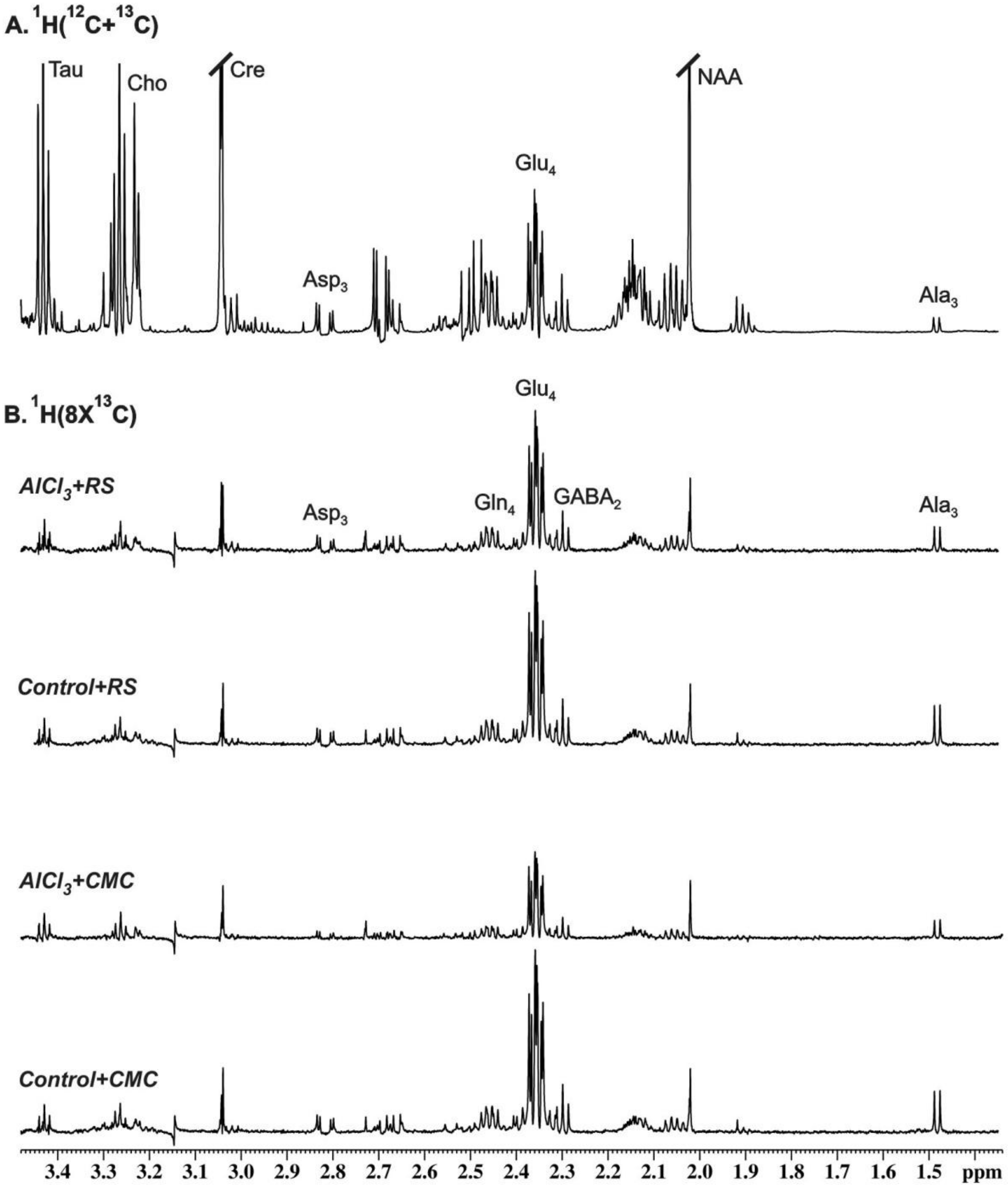

Supplement: Supplementary file 1 [file Presentation_1.pdf]
